# High-Resolution Observations of the Infrared Spectrum of Neutral Neon

**DOI:** 10.6028/jres.109.027

**Published:** 2004-06-01

**Authors:** Craig J. Sansonetti, Marion M. Blackwell, E. B. Saloman

**Affiliations:** National Institute of Standards and Technology, Gaithersburg, MD 20899

**Keywords:** atomic spectroscopy, Fourier transform spectroscopy, infrared, neon, wavelengths

## Abstract

We have observed the spectrum of neutral neon (Ne I) emitted by a microwave-excited electrodeless discharge lamp with the National Institute of Standards and Technology 2 m Fourier transform spectrometer. The spectra cover the regions 6929 Å to 11 000 Å with a resolution of 0.01 cm^−1^ and 11 000 Å to 47 589 Å with a resolution of 0.007 cm^−1^. We present a line list that includes more than 650 classified lines and provides an accurate and comprehensive description of the infrared spectrum. The response of the Fourier transform spectrometer was determined by using a radiometrically calibrated tungsten strip lamp, providing relative intensities that for moderate to strong lines are accurate to approximately 10 % over the entire range of the observations. The identities of many lines that were previously multiply classified are unambiguously resolved.

## 1. Introduction

Neon discharges are widely used in scientific, technical, and commercial applications. Despite the fact that Ne is frequently used as a buffer gas in sources for laboratory spectroscopy, no comprehensive description of its spectrum in the extraphotographic infrared region has appeared in the literature. Photographic spectra recorded with large grating spectrographs in the near infrared region have reasonably high resolution but extend to only about 12 000 Å [1]. At longer wavelengths, spectra obtained with infrared scanning instruments have resolution and accuracy that are very low by current standards [2,3].

The most complete line list for Ne at wavelengths longer than 11 000 Å was given by Humphreys [4] based on spectra recorded with a scanning 1 m grating spectrometer. The relative intensities in this list are experimental values from Humphreys’s grating observations, but the wavelengths are calculated from the Ne energy levels of Kaufman and Minnhagen [5]. Because of the low resolution and accuracy of the experimental spectra, many lines are multiply classified. In some cases as many as five classfications and calculated wavelengths are associated with a single feature in the observed spectrum. For such a case Ref. [4] provides no information about the relative intensities of these possible transitions in a high resolution spectrum, nor does it provide any basis for estimating the effective wavelength of the unresolved transitions at low resolution.

Measurements for 118 infrared lines of neon were reported by Chang et al. [6] based on hollow cathode spectra from the archives of the Fourier transform spectrometer of the National Solar Observatory at Kitt Peak National Observatory. These are the most accurate published measurements for neon in the infrared. Unfortunately, the work of Chang et al. has little value as a description of the spectrum since it gives no intensities and includes only selected lines.

In order to provide a comprehensive high-resolution description of the infrared spectrum of Ne, we made new observations with the National Institute of Standards and Technology (NIST) 2 m Fourier transform spectrometer (FTS). This is one component of a broader program of observations and compilations for the noble gases currently in progress at NIST. A more detailed description of this program and of our experimental observations for the infrared spectra of the noble gases has been given in Ref. [7]. We have also produced a complete compilation of transitions and energy levels for Ne I [8], which includes many data from the present work.

## 2. Experiment

**Fig. 1:**
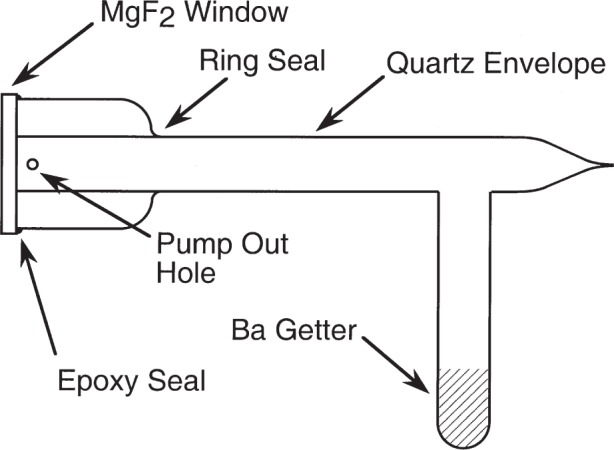
Design of the electrodeless discharge lamp used to observe the Ne I spectrum.

The spectrum was excited in a commercial sealed electrodeless discharge lamp filled for this work with Ne at a pressure of 200 Pa (1.5 Torr). The lamp design, illustrated in Fig. 1, is derived from that used by Wilkinson and Tanaka [9]. The lamp is equipped with a cemented magnesium fluoride window that permits viewing along the axis of the discharge. The double wall in the area of the window protects the epoxy seal from the discharge as demonstrated by Bass [10]. The lamp contains a barium getter in a side arm to trap impurities released from the walls during operation. Initial attempts to power the lamp using an Evenson cavity [11] were unsuccessful because we could not maintain stable tuning of the cavity over the several hours required for data acquisition. This resulted in large discontinuous variations in light output that were unacceptable for the FTS. We therefore turned to a cup-type antenna directed toward the side of the lamp near its midpoint to couple power to the discharge. This produced very stable excitation.

**Fig. 2:**
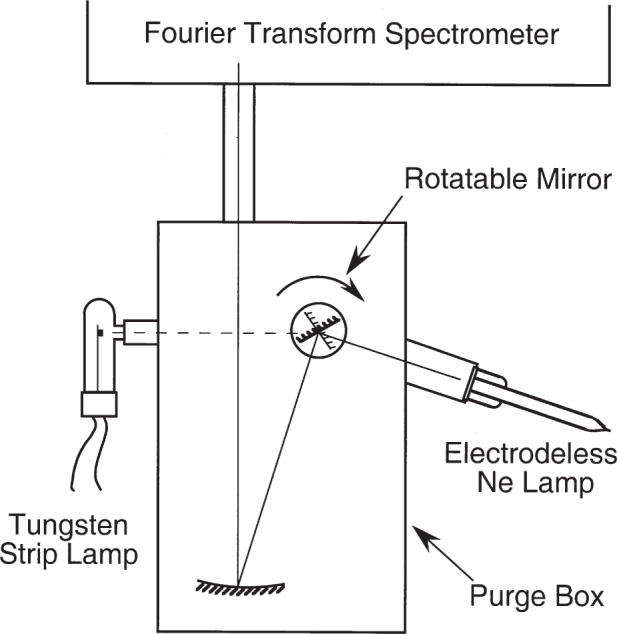
Schematic of the optical setup used to obtain radiometrically calibrated spectra.

Light from the electrodeless lamp was directed to the entrance aperture of the FTS through a path purged with dry air to avoid infrared absorption by atmospheric water vapor. The purged path incorporates a remotely-actuated rotating mirror and a concave mirror as shown in Fig. 2, permitting light from either the Ne lamp or a radiometric standard source to be imaged to the entrance aperture of the FTS. The radiometric standard source, a tungsten strip lamp with sapphire window, was used for calibration of the instrumental response.

**Fig. 3:**
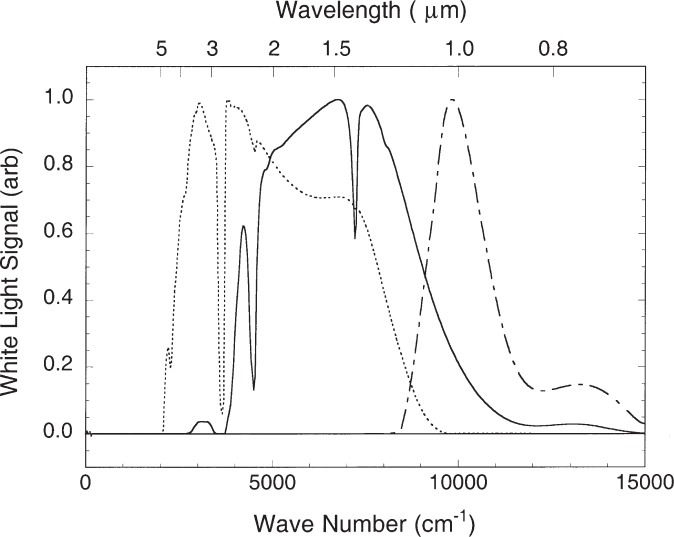
Spectrum of the tungsten strip lamp lamp as recorded with the three combinations of beamsplitter, filters, and detector used in this experiment. Each spectrum has been arbitrarily scaled to a maximum signal of 1.0. (dot-dash line—fused silica beamsplitter, Si photodiodes, 6500 Å high pass filter; solid line—fused silica beamsplitter, InSb detector, 6500 Å high pass filter; dotted line—CaF2 beamsplitter, InSb detector, antireflection coated Si filter)

Spectra were recorded with different combinations of beamsplitter, detector, and filter in three overlapping regions, covering the range from 7000 Å to 50 000 Å. The combinations used and resolution for each of these regions are summarized in Table 1. The instrumental resolution was 0.01 cm^−1^ for the shortest wavelength region, extending to 11 000 Å, and 0.007 cm^−1^ for the longer wavelength regions. This was sufficient to fully resolve all of the lines. Optical filters were used to eliminate the visible spectrum and scattered light from the He/Ne reference laser. Two detectors at the complementary outputs of the FTS were operated differentially to acquire the interferogram. Sixteen scans of the interferogram were coadded, representing a data acquisition time of about one hour for each spectrum. Six Ne spectra were recorded, two for each of the three spectral regions. Before or after each observation of the Ne lamp, a spectrum of the tungsten strip lamp was recorded. Representative spectra of this standard lamp for the three spectral regions are shown in Fig. 3. These calibration spectra were later used to correct the intensities in the Ne spectra for the response of the FTS, filters, and detectors.

The interferograms were transformed and the spectra were phase corrected and measured by using the interactive program Xgremlin, an X-windows implementation of the FTS analysis program GREMLIN [12] with graphical user interface and significantly enhanced capabilities that was developed by Griesmann at NIST. A brief description of Xgremlin is given in [13].

**Fig. 4:**
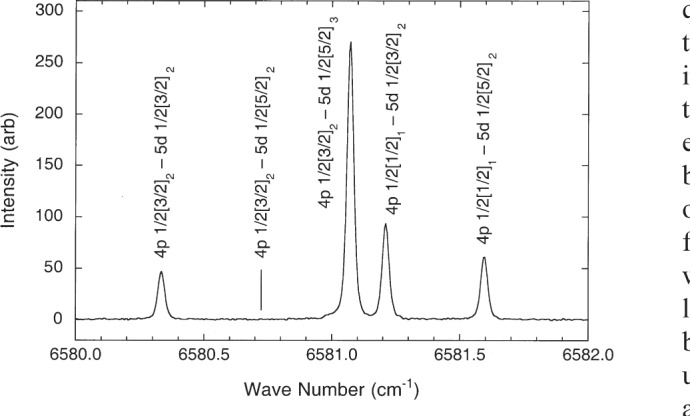
Ne spectral lines at 15 191 Å (6581 cm^−1^). The five classifications shown were assigned to a single experimentally observed feature in Ref. [4]. On this intensity scale the strongest line in the spectrum has intensity 100 000.

Ne has only small isotope shifts between the dominant ^20^Ne, which accounts for 91 % of the natural abundance, and ^22^Ne which makes up virtually all of the balance. Neither of these isotopes has magnetic hyperfine structure (hfs). Consequently, the observed lines in our Ne spectra were generally narrow and symmetric as illustrated by the lines in Fig. 4. Lines of this type were measured by fitting the observed profile with a Voigt function. A few lines had sufficiently large isotope shifts to display significant asymmetry. These lines were measured by interactively marking upper and lower wave-number limits that enclose the line and calculating the center of gravity of the spectrum between those limits. For all lines the integrated intensity under the line profile was determined.

## 3. Calibration and Data Processing

Spectra recorded with an FTS are linear in wave number to very high precision, but to obtain absolute accuracy of better than a few parts in 10^6^ it is necessary to correct the scale by a multiplicative constant derived from accurately known internal standard lines. For this purpose we used wave numbers calculated from optimized level values of the 2*p*^5^3*s*, 3*p*, 3*d*, and 4*s* configurations. Lines of these transition arrays have been recommended as secondary standards by the International Astronomical Union (IAU) [14]. Sixty-one lines of the 3*s*–3*p*, 3*p*–3*d*, and 3*p*–4*s* transition arrays were used for the calibration. Lines of the 3*d*–4*p* and 4*s*–4*p* transition arrays were not used because they gave results that were systematically inconsistent with the other calibration lines.

For each spectrum the lines were initially measured with respect to the uncorrected wave number scale. A correction factor was then determined by taking the unweighted average of individual correction factors calculated from each of the standard lines. Not all standard lines appeared in all spectra. For spectra covering the range 7000 Å to 11 000 Å about 40 standards were used and for the longer wavelength regions about 27 standards were used. In all cases the average correction was in the range 4 to 6 parts in 10^7^ and the standard deviation of the individual values was about 1 part in 10^7^.

The final wave number for each line was calculated from the corrected values as the unweighted average of the individual measurements. For most lines there were two to four observations. For a few lines there was only a single observation; for others there were as many as six. The uncertainty for each line was calculated as the quadrature sum of three terms: the calibration uncertainty as measured by the standard deviation of the individual line correction factors, the standard deviation of the multiple measurements of the line, and the estimated precision with which the line position could be measured in the spectra. For most lines of moderate or greater intensity the uncertainty is dominated by the first two terms. The third term is calculated as the line width divided by twice the signal-to-noise ratio at the line center [15]. It is included to insure that weak or broad lines are not assigned an unreasonably low uncertainty because of an accidentally high degree of agreement between a small number of measurements.

For each Ne spectrum the radiometric response of the combination of FTS, filters, and detectors was determined by recording the spectrum of the standard tungsten strip lamp with the same filters, detectors, and FTS observing parameters. This spectrum was compared to the previously calibrated output of the strip lamp to generate an instrumental response curve that was used to adjust the integrated intensities of the spectral lines to a uniform linear dependence on the number of photons detected. With this choice for the calibration, the ratios of intensities of lines with a common upper level give directly the branching ratios of the various decay paths.

After calibrated intensities had been obtained for each spectrum, lines of moderate intensity in the overlapping spectral regions were used to determine scaling factors that were applied to place all of the spectra on a common intensity scale. The intensities from the multiple observations of each line were then averaged. For the two spectra in the 13 000 Å to 50 000 Å region, intensities of the 11 lines between 9218 cm^−1^ and 9465 cm^−1^ were omitted from the intensity average because they differed systematically from the intensities of the same lines measured in the two shorter wavelength regions. These lines were at the extreme end of the long wavelength region where the instrumental response was very low. Finally, the entire set of average intensities was scaled to obtain values on a linear scale from 1 to 100 000.

In order to estimate the uncertainty of the intensities, we examined the ratio of the standard deviation to the average value for each of the approximately 600 lines for which more than one measurement was made. From this analysis we observed that the relative uncertainty of the intensities is about 10 % independent of intensity for lines with intensity of 100 or greater, 15 % for lines with intensities 10 to 100, and 25 % for lines weaker than 10. The analysis of uncertainties is summarized in Table 2, where we present the percentage of lines for which the relative standard deviation lies within 1, 2, and 3 times the stated uncertainty for each decade in the intensity. Based on this distribution, the stated uncertainties represent approximately a 90 % level of confidence. The statistics for the 10 000 to 100 000 intensity range reflect the fact that the few lines with intensities greater than 50 000, especially those that are self-reversed, are somewhat less reproducible than weaker lines.

## 4. Results

Results of this work are presented in Table 3. Virtually all lines previously reported in this spectral region have been observed. Only lines that could be reliably classified as transitions between Ne I levels are reported in the table. Impurity lines of Ar I, Xe I, O I, C I, and Hg I were identified and removed. Also removed were 23 weak lines that we were unable to identify. The strongest of these had intensity 57. All others were much weaker.

The intensities reported in the first column of Table 3 are on a scale linear in photon number as described above. Lines that were used as internal standards for calibration of the spectra are indicated by an S following the intensity. An asterisk in this position denotes a line that is multiply classified. In almost all cases multiply-classified lines are associated with the small intervals between pair-coupled levels in 5*p*^5^*nf* and 5*p*^5^*ng* configurations. These transitions are unresolvable in observation of the emission spectrum because their Doppler widths exceed the level separation. The many unresolved blends of transitions to 5*p*^5^*nd* levels in Ref. [4] have all been resolved in this work.

The observed wavelengths and their uncertainties are given in the second and third columns of Table 3. Wavelengths shorter than 20 000 Å are reported in standard air; wavelengths longer than 20 000 Å are vacuum values. The observed vacuum wave numbers were converted to standard air wavelengths by using the index of refraction of air as calculated from the three term formula of Peck and Reeder [16]. Uncertainties are reported at the one standard deviation level representing a 68 % confidence interval. The wavelengths have been rounded so that the uncertainty in the least significant digit does not exceed 20. Corresponding values of the vacuum wave number and its uncertainty are given in columns four and five.

The classification for each line is given in the remaining columns of Table 3. The configuration, term, and J value for the lower level is given first followed by the same information for the upper level. All level designations are given in the *J*_1_*l* coupling notation. The line identifications were initially made based on the Ne I level values reported by Kaufman and Minnhagen [5] and by Chang et al. [6]. A few identifications were later revised based on calculated transition probabilities and re-optimized level values determined in our comprehensive compilation of Ne I wavelengths and energy levels [8]. The classifications in Table 3 are fully consistent with Ref. [8].

## 5. Discussion

We have made new observations of the Ne I spectrum in the infrared region with high resolution and signal-to-noise ratio. Our results provide the first comprehensive experimental description of the spectrum in the extraphotographic infrared. Approximately 650 lines have been classified as transitions among previously reported Ne I levels. Most of the multiply-classified lines in the work of Humphreys [4] have now been resolved as described more fully in Ref. [7]. The lines shown in Fig. 4, for example, were reported as a single line with five classifications in Ref. [4].

Our newly measured wavelengths for all transitions to levels of the 5*p*^5^4*p* configuration in the 20 000 Å to 50 000 Å region are systematically shifted with respect to their calculated values based on the levels of Kaufman and Minnhagen [5] as we have previously described in Ref. [7]. The 5*p*^5^4*p* level values in Ref. [5] are consistent with values recommended by the IAU [14] as suitable for calculation of secondary wavelength standards. Our measurements indicate that the levels of this configuration must be shifted upward by approximately 0.0054 cm^−1^. A reoptimization of the 5*p*^5^4*p* levels based on all available data from the ultraviolet to the infrared has been made and confirms this shift [8].

## Figures and Tables

**Table 1:** Summary of observing parameters used in this experiment

Spectral region (Å)	Resolution (cm^−1^)	Beam splitter	Detector	Filter
7 000–11 000	0.01	Fused Silica	Si photodiode	High pass 6500 Å
11 000–35 000	0.007	Fused Silica	InSb	High pass 6500 Å
13 000–50 000	0.007	CaF2	InSb	AR coated silicon

**Table 2:** Distribution of the relative standard deviations of intensities for multiply determined lines with respect to multiples of the stated relative uncertainty

Intensity range	Number of multiply determined lines	Relative uncertainty *Δ*	Percentage of relative standard deviations		
			< *Δ*	< 2*Δ*	< 3*Δ*
10 000–100 000	40	10 %	85.0 %	95.0 %	97.5 %
1000–10 000	103	10 %	93.2 %	100.0 %	
100–1000	118	10 %	92.4 %	99.2 %	99.2 %
10–100	213	15 %	89.7 %	99.1 %	99.1 %
1–10	127	25 %	85.8 %	99.2 %	100.0 %

**Table 3:** Spectral lines of Ne I in the infrared

Intensity and comment^a^	Observed air wavelength (Å)	Uncertainty of observed wavelength (Å)	Observed wave number (cm^−1^)	Uncertainty of observed wave number (cm^−1^)	Classification						
					Configuration	Term	J		Configuration	Term	J
100000 r	6929.4686	0.0011	14427.141	0.002	2*p*^5^(^2^P^o^_1/2_)3*s*	^2^[1/2]^o^	1	—	2*p*^5^(^2^P^o^_3/2_)3*p*	^2^[3/2]	2
34000 S	7024.0498	0.0008	14232.8762	0.0016	2*p*^5^(^2^P^o^_1/2_)3*s*	^2^[1/2]^o^	1	—	2*p*^5^(^2^P^o^_3/2_)3*p*	^2^[3/2]	1
85000 r	7032.4153	0.0008	14215.9453	0.0016	2*p*^5^(^2^P^o^_3/2_)3*s*	^2^[3/2]^o^	2	—	2*p*^5^(^2^P^o^_3/2_)3*p*	^2^[1/2]	1
2200	7051.2922	0.0008	14177.8883	0.0016	2*p*^5^(^2^P^o^_3/2_)3*p*	^2^[1/2]	1	—	2*p*^5^(^2^P^o^_1/2_)3*d*	^2^[3/2]^o^	1
10000	7059.1072	0.0008	14162.1923	0.0016	2*p*^5^(^2^P^o^_3/2_)3*p*	^2^[1/2]	1	—	2*p*^5^(^2^P^o^_1/2_)3*d*	^2^[3/2]^o^	2
80	7064.762	0.003	14150.856	0.005	2*p*^5^(^2^P^o^_3/2_)3*p*	^2^[1/2]	1	—	2*p*^5^(^2^P^o^_1/2_)3*d*	^2^[5/2]^o^	2
110	7112.3075	0.0008	14056.2594	0.0016	2*p*^5^(^2^P^o^_1/2_)3*p*	^2^[1/2]	0	—	2*p*^5^(^2^P^o^_3/2_)4*d*	^2^[3/2]^o^	1
55	7138.5400	0.0011	14004.606	0.002	2*p*^5^(^2^P^o^_1/2_)3*p*	^2^[1/2]	0	—	2*p*^5^(^2^P^o^_3/2_)4*d*	^2^[1/2]^o^	1
77000 S	7173.9372	0.0009	13935.5059	0.0017	2*p*^5^(^2^P^o^_1/2_)3*s*	^2^[1/2]^o^	1	—	2*p*^5^(^2^P^o^_3/2_)3*p*	^2^[5/2]	2
77000 r	7245.1673	0.0008	13798.5013	0.0015	2*p*^5^(^2^P^o^_3/2_)3*s*	^2^[3/2]^o^	1	—	2*p*^5^(^2^P^o^_3/2_)3*p*	^2^[1/2]	1
89	7304.8422	0.0008	13685.7789	0.0015	2*p*^5^(^2^P^o^_1/2_)3*p*	^2^[1/2]	0	—	2*p*^5^(^2^P^o^_1/2_)5*s*	^2^[1/2]^o^	1
60000 S	7438.8970	0.0008	13439.1517	0.0015	2*p*^5^(^2^P^o^_1/2_)3*s*	^2^[1/2]^o^	0	—	2*p*^5^(^2^P^o^_3/2_)3*p*	^2^[1/2]	1
3100	7472.4380	0.0008	13378.8288	0.0015	2*p*^5^(^2^P^o^_3/2_)3*p*	^2^[1/2]	1	—	2*p*^5^(^2^P^o^_3/2_)3*d*	^2^[3/2]^o^	1
32000 S	7488.8712	0.0008	13349.4711	0.0015	2*p*^5^(^2^P^o^_3/2_)3*p*	^2^[1/2]	1	—	2*p*^5^(^2^P^o^_3/2_)3*d*	^2^[3/2]^o^	2
28000 S	7535.7738	0.0008	13266.3845	0.0014	2*p*^5^(^2^P^o^_3/2_)3*p*	^2^[1/2]	1	—	2*p*^5^(^2^P^o^_3/2_)3*d*	^2^[1/2]^o^	1
13000 S	7544.0435	0.0008	13251.8421	0.0014	2*p*^5^(^2^P^o^_3/2_)3*p*	^2^[1/2]	1	—	2*p*^5^(^2^P^o^_3/2_)3*d*	^2^[1/2]^o^	0
60	7724.6233	0.0009	12942.0533	0.0015	2*p*^5^(^2^P^o^_1/2_)3*p*	^2^[1/2]	0	—	2*p*^5^(^2^P^o^_3/2_)5*s*	^2^[3/2]^o^	1
56	7833.0281	0.0009	12762.9433	0.0014	2*p*^5^(^2^P^o^_3/2_)3*p*	^2^[5/2]	3	—	2*p*^5^(^2^P^o^_1/2_)3*d*	^2^[3/2]^o^	2
230	7839.0520	0.0009	12753.1358	0.0014	2*p*^5^(^2^P^o^_3/2_)3*p*	^2^[5/2]	3	—	2*p*^5^(^2^P^o^_1/2_)3*d*	^2^[5/2]^o^	3
7	7839.9855	0.0018	12751.617	0.003	2*p*^5^(^2^P^o^_3/2_)3*p*	^2^[5/2]	3	—	2*p*^5^(^2^P^o^_1/2_)3*d*	^2^[5/2]^o^	2
300	7927.1174	0.0009	12611.4570	0.0014	2*p*^5^(^2^P^o^_3/2_)3*p*	^2^[5/2]	2	—	2*p*^5^(^2^P^o^_1/2_)3*d*	^2^[3/2]^o^	1
1300	7936.9957	0.0009	12595.7610	0.0014	2*p*^5^(^2^P^o^_3/2_)3*p*	^2^[5/2]	2	—	2*p*^5^(^2^P^o^_1/2_)3*d*	^2^[3/2]^o^	2
7900 S	7943.1806	0.0009	12585.9533	0.0014	2*p*^5^(^2^P^o^_3/2_)3*p*	^2^[5/2]	2	—	2*p*^5^(^2^P^o^_1/2_)3*d*	^2^[5/2]^o^	3
200	7944.1404	0.0009	12584.4328	0.0014	2*p*^5^(^2^P^o^_3/2_)3*p*	^2^[5/2]	2	—	2*p*^5^(^2^P^o^_1/2_)3*d*	^2^[5/2]^o^	2
5700	8082.4578	0.0009	12369.0729	0.0014	2*p*^5^(^2^P^o^_1/2_)3*s*	^2^[1/2]^o^	1	—	2*p*^5^(^2^P^o^_3/2_)3*p*	^2^[1/2]	1
3800	8118.5495	0.0009	12314.0854	0.0013	2*p*^5^(^2^P^o^_3/2_)3*p*	^2^[3/2]	1	—	2*p*^5^(^2^P^o^_1/2_)3*d*	^2^[3/2]^o^	1
1200	8128.9110	0.0009	12298.3893	0.0013	2*p*^5^(^2^P^o^_3/2_)3*p*	^2^[3/2]	1	—	2*p*^5^(^2^P^o^_1/2_)3*d*	^2^[3/2]^o^	2
17000 S	8136.4054	0.0009	12287.0614	0.0013	2*p*^5^(^2^P^o^_3/2_)3*p*	^2^[3/2]	1	—	2*p*^5^(^2^P^o^_1/2_)3*d*	^2^[5/2]^o^	2
310	8248.6826	0.0009	12119.8164	0.0013	2*p*^5^(^2^P^o^_3/2_)3*p*	^2^[3/2]	2	—	2*p*^5^(^2^P^o^_1/2_)3*d*	^2^[3/2]^o^	1
3300	8259.3791	0.0009	12104.1205	0.0013	2*p*^5^(^2^P^o^_3/2_)3*p*	^2^[3/2]	2	—	2*p*^5^(^2^P^o^_1/2_)3*d*	^2^[3/2]^o^	2
7200	8266.0769	0.0009	12094.3129	0.0013	2*p*^5^(^2^P^o^_3/2_)3*p*	^2^[3/2]	2	—	2*p*^5^(^2^P^o^_1/2_)3*d*	^2^[5/2]^o^	3
990	8267.1161	0.0009	12092.7925	0.0013	2*p*^5^(^2^P^o^_3/2_)3*p*	^2^[3/2]	2	—	2*p*^5^(^2^P^o^_1/2_)3*d*	^2^[5/2]^o^	2
29000 S	8300.3246	0.0009	12044.4111	0.0013	2*p*^5^(^2^P^o^_3/2_)3*p*	^2^[5/2]	3	—	2*p*^5^(^2^P^o^_3/2_)3*d*	^2^[5/2]^o^	3
1900	8301.5570	0.0009	12042.6230	0.0013	2*p*^5^(^2^P^o^_3/2_)3*p*	^2^[5/2]	3	—	2*p*^5^(^2^P^o^_3/2_)3*d*	^2^[5/2]^o^	2
4600	8365.7462	0.0009	11950.2222	0.0013	2*p*^5^(^2^P^o^_3/2_)3*p*	^2^[5/2]	3	—	2*p*^5^(^2^P^o^_3/2_)3*d*	^2^[3/2]^o^	2
6600	8376.3590	0.0009	11935.0813	0.0013	2*p*^5^(^2^P^o^_3/2_)3*p*	^2^[5/2]	3	—	2*p*^5^(^2^P^o^_3/2_)3*d*	^2^[7/2]^o^	3
76000 S	8377.6060	0.0009	11933.3048	0.0013	2*p*^5^(^2^P^o^_3/2_)3*p*	^2^[5/2]	3	—	2*p*^5^(^2^P^o^_3/2_)3*d*	^2^[7/2]^o^	4
2700	8417.1597	0.0009	11877.2284	0.0013	2*p*^5^(^2^P^o^_3/2_)3*p*	^2^[5/2]	2	—	2*p*^5^(^2^P^o^_3/2_)3*d*	^2^[5/2]^o^	3
26000 S	8418.4269	0.0009	11875.4406	0.0013	2*p*^5^(^2^P^o^_3/2_)3*p*	^2^[5/2]	2	—	2*p*^5^(^2^P^o^_3/2_)3*d*	^2^[5/2]^o^	2
3700	8463.3565	0.0009	11812.3974	0.0013	2*p*^5^(^2^P^o^_3/2_)3*p*	^2^[5/2]	2	—	2*p*^5^(^2^P^o^_3/2_)3*d*	^2^[3/2]^o^	1
1300	8484.4432	0.0009	11783.0397	0.0013	2*p*^5^(^2^P^o^_3/2_)3*p*	^2^[5/2]	2	—	2*p*^5^(^2^P^o^_3/2_)3*d*	^2^[3/2]^o^	2
69000 S	8495.3595	0.0009	11767.8989	0.0013	2*p*^5^(^2^P^o^_3/2_)3*p*	^2^[5/2]	2	—	2*p*^5^(^2^P^o^_3/2_)3*d*	^2^[7/2]^o^	3
1600	8544.6953	0.0009	11699.9532	0.0013	2*p*^5^(^2^P^o^_3/2_)3*p*	^2^[5/2]	2	—	2*p*^5^(^2^P^o^_3/2_)3*d*	^2^[1/2]^o^	1
2900	8571.3528	0.0009	11663.5656	0.0013	2*p*^5^(^2^P^o^_1/2_)3*p*	^2^[3/2]	1	—	2*p*^5^(^2^P^o^_1/2_)3*d*	^2^[3/2]^o^	1
1600	8582.9031	0.0009	11647.8696	0.0013	2*p*^5^(^2^P^o^_1/2_)3*p*	^2^[3/2]	1	—	2*p*^5^(^2^P^o^_1/2_)3*d*	^2^[3/2]^o^	2
41000 S	8591.2585	0.0009	11636.5416	0.0013	2*p*^5^(^2^P^o^_1/2_)3*p*	^2^[3/2]	1	—	2*p*^5^(^2^P^o^_1/2_)3*d*	^2^[5/2]^o^	2
35000 S	8634.6471	0.0009	11578.0689	0.0013	2*p*^5^(^2^P^o^_3/2_)3*p*	^2^[3/2]	1	—	2*p*^5^(^2^P^o^_3/2_)3*d*	^2^[5/2]^o^	2
740	8635.3177	0.0010	11577.1698	0.0013	2*p*^5^(^2^P^o^_1/2_)3*p*	^2^[3/2]	2	—	2*p*^5^(^2^P^o^_1/2_)3*d*	^2^[3/2]^o^	1
6000	8647.0412	0.0009	11561.4737	0.0013	2*p*^5^(^2^P^o^_1/2_)3*p*	^2^[3/2]	2	—	2*p*^5^(^2^P^o^_1/2_)3*d*	^2^[3/2]^o^	2
64000 S	8654.3828	0.0009	11551.6661	0.0013	2*p*^5^(^2^P^o^_1/2_)3*p*	^2^[3/2]	2	—	2*p*^5^(^2^P^o^_1/2_)3*d*	^2^[5/2]^o^	3
7600	8655.5220	0.0009	11550.1457	0.0013	2*p*^5^(^2^P^o^_1/2_)3*p*	^2^[3/2]	2	—	2*p*^5^(^2^P^o^_1/2_)3*d*	^2^[5/2]^o^	2
13000 S	8679.4936	0.0010	11518.2459	0.0013	2*p*^5^(^2^P^o^_3/2_)3*p*	^2^[1/2]	0	—	2*p*^5^(^2^P^o^_1/2_)3*d*	^2^[3/2]^o^	1
15000 S	8681.9208	0.0009	11515.0258	0.0013	2*p*^5^(^2^P^o^_3/2_)3*p*	^2^[3/2]	1	—	2*p*^5^(^2^P^o^_3/2_)3*d*	^2^[3/2]^o^	1
2900	8704.1122	0.0010	11485.6680	0.0013	2*p*^5^(^2^P^o^_3/2_)3*p*	^2^[3/2]	1	—	2*p*^5^(^2^P^o^_3/2_)3*d*	^2^[3/2]^o^	2
160	8767.536	0.004	11402.581	0.005	2*p*^5^(^2^P^o^_3/2_)3*p*	^2^[3/2]	1	—	2*p*^5^(^2^P^o^_3/2_)3*d*	^2^[1/2]^o^	1
10000 S	8771.6575	0.0010	11397.2240	0.0012	2*p*^5^(^2^P^o^_1/2_)3*p*	^2^[1/2]	1	—	2*p*^5^(^2^P^o^_1/2_)3*d*	^2^[3/2]^o^	1
2100	8778.7322	0.0010	11388.0391	0.0012	2*p*^5^(^2^P^o^_3/2_)3*p*	^2^[3/2]	1	—	2*p*^5^(^2^P^o^_3/2_)3*d*	^2^[1/2]^o^	0
57000 S	8780.6222	0.0010	11385.5880	0.0012	2*p*^5^(^2^P^o^_3/2_)3*p*	^2^[3/2]	2	—	2*p*^5^(^2^P^o^_3/2_)3*d*	^2^[5/2]^o^	3
230	8782.0015	0.0010	11383.7997	0.0013	2*p*^5^(^2^P^o^_3/2_)3*p*	^2^[3/2]	2	—	2*p*^5^(^2^P^o^_3/2_)3*d*	^2^[5/2]^o^	2
3000 S	8783.7543	0.0010	11381.5280	0.0012	2*p*^5^(^2^P^o^_1/2_)3*p*	^2^[1/2]	1	—	2*p*^5^(^2^P^o^_1/2_)3*d*	^2^[3/2]^o^	2
260	8792.5056	0.0010	11370.1999	0.0012	2*p*^5^(^2^P^o^_1/2_)3*p*	^2^[1/2]	1	—	2*p*^5^(^2^P^o^_1/2_)3*d*	^2^[5/2]^o^	2
550	8830.9067	0.0010	11320.7570	0.0012	2*p*^5^(^2^P^o^_3/2_)3*p*	^2^[3/2]	2	—	2*p*^5^(^2^P^o^_3/2_)3*d*	^2^[3/2]^o^	1
27000 S	8853.8672	0.0010	11291.3992	0.0012	2*p*^5^(^2^P^o^_3/2_)3*p*	^2^[3/2]	2	—	2*p*^5^(^2^P^o^_3/2_)3*d*	^2^[3/2]^o^	2
2100	8865.3058	0.0010	11276.8304	0.0012	2*p*^5^(^2^P^o^_3/2_)3*p*	^2^[1/2]	1	—	2*p*^5^(^2^P^o^_1/2_)4*s*	^2^[1/2]^o^	1
15000 S	8865.7556	0.0010	11276.2583	0.0012	2*p*^5^(^2^P^o^_3/2_)3*p*	^2^[3/2]	2	—	2*p*^5^(^2^P^o^_3/2_)3*d*	^2^[7/2]^o^	3
12	8892.2315	0.0017	11242.684	0.002	2*p*^5^(^2^P^o^_3/2_)4*s*	^2^[3/2]^o^	2	—	2*p*^5^(^2^P^o^_3/2_)6*p*	^2^[3/2]	2
6400 S	8919.5007	0.0010	11208.3127	0.0012	2*p*^5^(^2^P^o^_3/2_)3*p*	^2^[3/2]	2	—	2*p*^5^(^2^P^o^_3/2_)3*d*	^2^[1/2]^o^	1
18	8929.2503	0.0012	11196.0746	0.0015	2*p*^5^(^2^P^o^_3/2_)4*s*	^2^[3/2]^o^	2	—	2*p*^5^(^2^P^o^_3/2_)6*p*	^2^[5/2]	3
8	8941.5133	0.0014	11180.7196	0.0018	2*p*^5^(^2^P^o^_3/2_)4*s*	^2^[3/2]^o^	1	—	2*p*^5^(^2^P^o^_3/2_)6*p*	^2^[1/2]	0
4	8962.328	0.004	11154.753	0.005	2*p*^5^(^2^P^o^_1/2_)4*s*	^2^[1/2]^o^	1	—	2*p*^5^(^2^P^o^_1/2_)6*p*	^2^[1/2]	0
1800 S	8988.5564	0.0010	11122.2037	0.0012	2*p*^5^(^2^P^o^_3/2_)3*p*	^2^[1/2]	1	—	2*p*^5^(^2^P^o^_1/2_)4*s*	^2^[1/2]^o^	0
9	9036.9985	0.0016	11062.5843	0.0020	2*p*^5^(^2^P^o^_1/2_)4*s*	^2^[1/2]^o^	1	—	2*p*^5^(^2^P^o^_1/2_)6*p*	^2^[3/2]	2
4	9049.086	0.004	11047.808	0.005	2*p*^5^(^2^P^o^_3/2_)4*s*	^2^[3/2]^o^	1	—	2*p*^5^(^2^P^o^_3/2_)6*p*	^2^[3/2]	2
3	9052.424	0.008	11043.734	0.010	2*p*^5^(^2^P^o^_1/2_)4*s*	^2^[1/2]^o^	1	—	2*p*^5^(^2^P^o^_1/2_)6*p*	^2^[1/2]	1
7	9052.642	0.002	11043.468	0.003	2*p*^5^(^2^P^o^_3/2_)4*s*	^2^[3/2]^o^	1	—	2*p*^5^(^2^P^o^_3/2_)6*p*	^2^[3/2]	1
5	9073.033	0.002	11018.649	0.002	2*p*^5^(^2^P^o^_3/2_)4*s*	^2^[3/2]^o^	1	—	2*p*^5^(^2^P^o^_3/2_)6*p*	^2^[5/2]	2
12000 S	9148.6720	0.0010	10927.5491	0.0012	2*p*^5^(^2^P^o^_1/2_)3*p*	^2^[3/2]	1	—	2*p*^5^(^2^P^o^_3/2_)3*d*	^2^[5/2]^o^	2
8900 S	9201.7588	0.0010	10864.5060	0.0012	2*p*^5^(^2^P^o^_1/2_)3*p*	^2^[3/2]	1	—	2*p*^5^(^2^P^o^_3/2_)3*d*	^2^[3/2]^o^	1
6000 S	9220.0598	0.0010	10842.9411	0.0012	2*p*^5^(^2^P^o^_1/2_)3*p*	^2^[3/2]	2	—	2*p*^5^(^2^P^o^_3/2_)3*d*	^2^[5/2]^o^	3
2200	9221.5802	0.0010	10841.1533	0.0012	2*p*^5^(^2^P^o^_1/2_)3*p*	^2^[3/2]	2	—	2*p*^5^(^2^P^o^_3/2_)3*d*	^2^[5/2]^o^	2
1800	9226.6910	0.0010	10835.1483	0.0012	2*p*^5^(^2^P^o^_1/2_)3*p*	^2^[3/2]	1	—	2*p*^5^(^2^P^o^_3/2_)3*d*	^2^[3/2]^o^	2
910	9275.5191	0.0010	10778.1102	0.0012	2*p*^5^(^2^P^o^_1/2_)3*p*	^2^[3/2]	2	—	2*p*^5^(^2^P^o^_3/2_)3*d*	^2^[3/2]^o^	1
7700 S	9300.8532	0.0010	10748.7524	0.0012	2*p*^5^(^2^P^o^_1/2_)3*p*	^2^[3/2]	2	—	2*p*^5^(^2^P^o^_3/2_)3*d*	^2^[3/2]^o^	2
830	9310.5833	0.0010	10737.5193	0.0012	2*p*^5^(^2^P^o^_1/2_)3*p*	^2^[3/2]	1	—	2*p*^5^(^2^P^o^_3/2_)3*d*	^2^[1/2]^o^	0
2700	9313.9731	0.0010	10733.6115	0.0012	2*p*^5^(^2^P^o^_1/2_)3*p*	^2^[3/2]	2	—	2*p*^5^(^2^P^o^_3/2_)3*d*	^2^[7/2]^o^	3
6900 S	9326.5072	0.0010	10719.1864	0.0012	2*p*^5^(^2^P^o^_3/2_)3*p*	^2^[1/2]	0	—	2*p*^5^(^2^P^o^_3/2_)3*d*	^2^[3/2]^o^	1
1500	9373.3079	0.0010	10665.6659	0.0012	2*p*^5^(^2^P^o^_1/2_)3*p*	^2^[3/2]	2	—	2*p*^5^(^2^P^o^_3/2_)3*d*	^2^[1/2]^o^	1
7	9377.2276	0.0013	10661.2077	0.0014	2*p*^5^(^2^P^o^_1/2_)3*p*	^2^[1/2]	1	—	2*p*^5^(^2^P^o^_3/2_)3*d*	^2^[5/2]^o^	2
4800 S	9425.3797	0.0010	10606.7422	0.0012	2*p*^5^(^2^P^o^_3/2_)3*p*	^2^[1/2]	0	—	2*p*^5^(^2^P^o^_3/2_)3*d*	^2^[1/2]^o^	1
66	9433.0082	0.0010	10598.1645	0.0012	2*p*^5^(^2^P^o^_1/2_)3*p*	^2^[1/2]	1	—	2*p*^5^(^2^P^o^_3/2_)3*d*	^2^[3/2]^o^	1
2800	9459.2110	0.0010	10568.8068	0.0012	2*p*^5^(^2^P^o^_1/2_)3*p*	^2^[1/2]	1	—	2*p*^5^(^2^P^o^_3/2_)3*d*	^2^[3/2]^o^	2
5000 S	9486.6825	0.0010	10538.2018	0.0012	2*p*^5^(^2^P^o^_3/2_)3*p*	^2^[1/2]	1	—	2*p*^5^(^2^P^o^_3/2_)4*s*	^2^[3/2]^o^	1
6100 S	9534.1640	0.0010	10485.7203	0.0011	2*p*^5^(^2^P^o^_1/2_)3*p*	^2^[1/2]	1	—	2*p*^5^(^2^P^o^_3/2_)3*d*	^2^[1/2]^o^	1
2800 S	9547.4052	0.0010	10471.1778	0.0011	2*p*^5^(^2^P^o^_1/2_)3*p*	^2^[1/2]	1	—	2*p*^5^(^2^P^o^_3/2_)3*d*	^2^[1/2]^o^	0
2	9574.002	0.003	10442.089	0.003	2*p*^5^(^2^P^o^_1/2_)4*s*	^2^[1/2]^o^	1	—	2*p*^5^(^2^P^o^_3/2_)6*p*	^2^[1/2]	0
18000 S	9665.4200	0.0011	10343.3251	0.0011	2*p*^5^(^2^P^o^_3/2_)3*p*	^2^[1/2]	1	—	2*p*^5^(^2^P^o^_3/2_)4*s*	^2^[3/2]^o^	2
3	9823.453	0.009	10176.929	0.010	2*p*^5^(^2^P^o^_3/2_)3*d*	^2^[1/2]^o^	0	—	2*p*^5^(^2^P^o^_3/2_)7*f*	^2^[3/2]	1
13 *	9837.507	0.012	10162.391	0.012	2*p*^5^(^2^P^o^_3/2_)3*d*	^2^[1/2]^o^	1	—	2*p*^5^(^2^P^o^_3/2_)7*f*	^2^[3/2]	1
13 *	9837.507	0.012	10162.391	0.012	2*p*^5^(^2^P^o^_3/2_)3*d*	^2^[1/2]^o^	1	—	2*p*^5^(^2^P^o^_3/2_)7*f*	^2^[3/2]	2
30 a*	9900.594	0.010	10097.636	0.011	2*p*^5^(^2^P^o^_3/2_)3*d*	^2^[7/2]^o^	4	—	2*p*^5^(^2^P^o^_3/2_)7*f*	^2^[9/2]	4
30 a*	9900.594	0.010	10097.636	0.011	2*p*^5^(^2^P^o^_3/2_)3*d*	^2^[7/2]^o^	4	—	2*p*^5^(^2^P^o^_3/2_)7*f*	^2^[9/2]	5
25 a	9902.337	0.010	10095.858	0.010	2*p*^5^(^2^P^o^_3/2_)3*d*	^2^[7/2]^o^	3	—	2*p*^5^(^2^P^o^_3/2_)7*f*	^2^[9/2]	4
13	9915.195	0.006	10082.766	0.006	2*p*^5^(^2^P^o^_3/2_)3*d*	^2^[3/2]^o^	2	—	2*p*^5^(^2^P^o^_3/2_)7*f*	^2^[5/2]	3
6 *	9918.602	0.008	10079.303	0.008	2*p*^5^(^2^P^o^_3/2_)3*d*	^2^[3/2]^o^	2	—	2*p*^5^(^2^P^o^_3/2_)7*f*	^2^[3/2]	1
6 *	9918.602	0.008	10079.303	0.008	2*p*^5^(^2^P^o^_3/2_)3*d*	^2^[3/2]^o^	2	—	2*p*^5^(^2^P^o^_3/2_)7*f*	^2^[3/2]	2
10	9936.853	0.003	10060.790	0.003	2*p*^5^(^2^P^o^_1/2_)3*d*	^2^[5/2]^o^	2	—	2*p*^5^(^2^P^o^_1/2_)7*f*	^2^[7/2]	3
16	9938.352	0.002	10059.273	0.002	2*p*^5^(^2^P^o^_1/2_)3*d*	^2^[5/2]^o^	3	—	2*p*^5^(^2^P^o^_1/2_)7*f*	^2^[7/2]	4
9	9944.140	0.007	10053.418	0.007	2*p*^5^(^2^P^o^_3/2_)3*d*	^2^[3/2]^o^	1	—	2*p*^5^(^2^P^o^_3/2_)7*f*	^2^[5/2]	2
4 *	9945.058	0.004	10052.489	0.004	2*p*^5^(^2^P^o^_3/2_)3*d*	^2^[3/2]^o^	2	—	2*p*^5^(^2^P^o^_1/2_)6*f*	^2^[5/2]	2
4 *	9945.058	0.004	10052.489	0.004	2*p*^5^(^2^P^o^_3/2_)3*d*	^2^[3/2]^o^	2	—	2*p*^5^(^2^P^o^_1/2_)6*f*	^2^[5/2]	3
9	9948.061	0.020	10049.45	0.02	2*p*^5^(^2^P^o^_1/2_)3*d*	^2^[3/2]^o^	2	—	2*p*^5^(^2^P^o^_1/2_)7*f*	^2^[5/2]	3
7	9963.605	0.006	10033.777	0.006	2*p*^5^(^2^P^o^_1/2_)3*d*	^2^[3/2]^o^	1	—	2*p*^5^(^2^P^o^_1/2_)7*f*	^2^[5/2]	2
13	10005.600	0.004	9991.664	0.004	2*p*^5^(^2^P^o^_3/2_)3*d*	^2^[5/2]^o^	2	—	2*p*^5^(^2^P^o^_3/2_)7*f*	^2^[7/2]	3
20	10007.385	0.004	9989.882	0.004	2*p*^5^(^2^P^o^_3/2_)3*d*	^2^[5/2]^o^	3	—	2*p*^5^(^2^P^o^_3/2_)7*f*	^2^[7/2]	4
5	10008.685	0.011	9988.585	0.011	2*p*^5^(^2^P^o^_3/2_)3*d*	^2^[5/2]^o^	3	—	2*p*^5^(^2^P^o^_3/2_)7*f*	^2^[5/2]	3
2	10210.835	0.004	9790.835	0.004	2*p*^5^(^2^P^o^_3/2_)4*s*	^2^[3/2]^o^	1	—	2*p*^5^(^2^P^o^_1/2_)5*p*	^2^[1/2]	0
4	10224.659	0.002	9777.597	0.002	2*p*^5^(^2^P^o^_3/2_)4*s*	^2^[3/2]^o^	2	—	2*p*^5^(^2^P^o^_1/2_)5*p*	^2^[3/2]	2
16	10245.7132	0.0013	9757.5052	0.0013	2*p*^5^(^2^P^o^_3/2_)4*s*	^2^[3/2]^o^	2	—	2*p*^5^(^2^P^o^_1/2_)5*p*	^2^[1/2]	1
420	10295.4162	0.0011	9710.3992	0.0011	2*p*^5^(^2^P^o^_3/2_)3*p*	^2^[5/2]	2	—	2*p*^5^(^2^P^o^_1/2_)4*s*	^2^[1/2]^o^	1
13 *	10432.5909	0.0017	9582.7207	0.0016	2*p*^5^(^2^P^o^_3/2_)4*p*	^2^[5/2]	2	—	2*p*^5^(^2^P^o^_3/2_)10*s*	^2^[3/2]^o^	1
13 *	10432.5909	0.0017	9582.7207	0.0016	2*p*^5^(^2^P^o^_3/2_)4*s*	^2^[3/2]^o^	1	—	2*p*^5^(^2^P^o^_1/2_)5*p*	^2^[3/2]	2
8000 S	10562.4089	0.0012	9464.9440	0.0011	2*p*^5^(^2^P^o^_1/2_)3*p*	^2^[1/2]	0	—	2*p*^5^(^2^P^o^_1/2_)3*d*	^2^[3/2]^o^	1
780 S	10620.6637	0.0015	9413.0285	0.0014	2*p*^5^(^2^P^o^_3/2_)3*p*	^2^[3/2]	1	—	2*p*^5^(^2^P^o^_1/2_)4*s*	^2^[1/2]^o^	1
19	10673.870	0.003	9366.107	0.003	2*p*^5^(^2^P^o^_3/2_)3*d*	^2^[1/2]^o^	0	—	2*p*^5^(^2^P^o^_3/2_)6*f*	^2^[3/2]	1
55 *	10690.457	0.005	9351.576	0.004	2*p*^5^(^2^P^o^_3/2_)3*d*	^2^[1/2]^o^	1	—	2*p*^5^(^2^P^o^_3/2_)6*f*	^2^[3/2]	2
55 *	10690.457	0.005	9351.576	0.004	2*p*^5^(^2^P^o^_3/2_)3*d*	^2^[1/2]^o^	1	—	2*p*^5^(^2^P^o^_3/2_)6*f*	^2^[3/2]	1
15	10758.204	0.013	9292.686	0.011	2*p*^5^(^2^P^o^_3/2_)3*d*	^2^[7/2]^o^	4	—	2*p*^5^(^2^P^o^_3/2_)6*f*	^2^[7/2]	4
14 *	10760.270	0.004	9290.902	0.003	2*p*^5^(^2^P^o^_3/2_)3*d*	^2^[7/2]^o^	3	—	2*p*^5^(^2^P^o^_3/2_)6*f*	^2^[7/2]	4
14 *	10760.270	0.004	9290.902	0.003	2*p*^5^(^2^P^o^_3/2_)3*d*	^2^[7/2]^o^	3	—	2*p*^5^(^2^P^o^_3/2_)6*f*	^2^[7/2]	3
150	10764.023	0.003	9287.662	0.003	2*p*^5^(^2^P^o^_3/2_)3*d*	^2^[7/2]^o^	4	—	2*p*^5^(^2^P^o^_3/2_)6*f*	^2^[9/2]	5
110	10766.087	0.003	9285.882	0.003	2*p*^5^(^2^P^o^_3/2_)3*d*	^2^[7/2]^o^	3	—	2*p*^5^(^2^P^o^_3/2_)6*f*	^2^[9/2]	4
69	10780.531	0.004	9273.441	0.003	2*p*^5^(^2^P^o^_3/2_)3*d*	^2^[3/2]^o^	2	—	2*p*^5^(^2^P^o^_3/2_)6*f*	^2^[5/2]	3
16 *	10786.286	0.003	9268.493	0.002	2*p*^5^(^2^P^o^_3/2_)3*d*	^2^[3/2]^o^	2	—	2*p*^5^(^2^P^o^_3/2_)6*f*	^2^[3/2]	2
16 *	10786.286	0.003	9268.493	0.002	2*p*^5^(^2^P^o^_3/2_)3*d*	^2^[3/2]^o^	2	—	2*p*^5^(^2^P^o^_3/2_)6*f*	^2^[3/2]	1
2	10790.862	0.004	9264.562	0.004	2*p*^5^(^2^P^o^_1/2_)4*p*	^2^[1/2]	1	—	2*p*^5^(^2^P^o^_3/2_)12*s*	^2^[3/2]^o^	1
6100 S	10798.0427	0.0013	9258.4014	0.011	2*p*^5^(^2^P^o^_3/2_)3*p*	^2^[3/2]	1	—	2*p*^5^(^2^P^o^_1/2_)4*s*	^2^[1/2]^o^	0
54	10806.358	0.006	9251.278	0.005	2*p*^5^(^2^P^o^_1/2_)3*d*	^2^[5/2]^o^	2	—	2*p*^5^(^2^P^o^_1/2_)6*f*	^2^[7/2]	3
74	10808.128	0.005	9249.762	0.004	2*p*^5^(^2^P^o^_1/2_)3*d*	^2^[5/2]^o^	3	—	2*p*^5^(^2^P^o^_1/2_)6*f*	^2^[7/2]	4
40	10814.755	0.007	9244.095	0.006	2*p*^5^(^2^P^o^_3/2_)3*d*	^2^[3/2]^o^	1	—	2*p*^5^(^2^P^o^_3/2_)6*f*	^2^[5/2]	2
92	10819.819	0.006	9239.768	0.005	2*p*^5^(^2^P^o^_1/2_)3*d*	^2^[3/2]^o^	2	—	2*p*^5^(^2^P^o^_1/2_)6*f*	^2^[5/2]	3
37	10838.2180	0.0014	9224.0824	0.0012	2*p*^5^(^2^P^o^_1/2_)3*d*	^2^[3/2]^o^	1	—	2*p*^5^(^2^P^o^_1/2_)6*f*	^2^[5/2]	2
9400 S	10844.4762	0.0013	9218.7593	0.0011	2*p*^5^(^2^P^o^_3/2_)3*p*	^2^[3/2]	2	—	2*p*^5^(^2^P^o^_1/2_)4*s*	^2^[1/2]^o^	1
58	10886.277	0.008	9183.362	0.007	2*p*^5^(^2^P^o^_3/2_)3*d*	^2^[5/2]^o^	2	—	2*p*^5^(^2^P^o^_3/2_)6*f*	^2^[7/2]	3
85	10888.392	0.008	9181.577	0.007	2*p*^5^(^2^P^o^_3/2_)3*d*	^2^[5/2]^o^	3	—	2*p*^5^(^2^P^o^_3/2_)6*f*	^2^[7/2]	4
25	10891.151	0.006	9179.252	0.005	2*p*^5^(^2^P^o^_3/2_)3*d*	^2^[5/2]^o^	3	—	2*p*^5^(^2^P^o^_3/2_)6*f*	^2^[5/2]	3
78	11020.8794	0.0015	9071.2017	0.0012	2*p*^5^(^2^P^o^_3/2_)4*s*	^2^[3/2]^o^	1	—	2*p*^5^(^2^P^o^_3/2_)5*p*	^2^[1/2]	0
64	11044.0002	0.0015	9052.2110	0.0012	2*p*^5^(^2^P^o^_1/2_)4*s*	^2^[1/2]^o^	1	—	2*p*^5^(^2^P^o^_1/2_)5*p*	^2^[1/2]	0
200	11049.7221	0.0014	9047.5235	0.0011	2*p*^5^(^2^P^o^_3/2_)4*s*	^2^[3/2]^o^	2	—	2*p*^5^(^2^P^o^_3/2_)5*p*	^2^[3/2]	2
29	11060.808	0.002	9038.4556	0.0020	2*p*^5^(^2^P^o^_3/2_)4*s*	^2^[3/2]^o^	2	—	2*p*^5^(^2^P^o^_3/2_)5*p*	^2^[3/2]	1
73	11120.2780	0.0016	8990.1190	0.0013	2*p*^5^(^2^P^o^_3/2_)4*s*	^2^[3/2]^o^	2	—	2*p*^5^(^2^P^o^_3/2_)5*p*	^2^[5/2]	2
49	11134.5095	0.0019	8978.6283	0.0016	2*p*^5^(^2^P^o^_1/2_)4*s*	^2^[1/2]^o^	0	—	2*p*^5^(^2^P^o^_1/2_)5*p*	^2^[1/2]	1
55	11138.4329	0.0015	8975.4657	0.0012	2*p*^5^(^2^P^o^_1/2_)4*s*	^2^[1/2]^o^	0	—	2*p*^5^(^2^P^o^_1/2_)5*p*	^2^[3/2]	1
26000 S	11143.0198	0.0013	8971.7710	0.0011	2*p*^5^(^2^P^o^_3/2_)3*p*	^2^[5/2]	2	—	2*p*^5^(^2^P^o^_3/2_)4*s*	^2^[3/2]^o^	1
270	11160.2133	0.0014	8957.9491	0.0011	2*p*^5^(^2^P^o^_3/2_)4*s*	^2^[3/2]^o^	2	—	2*p*^5^(^2^P^o^_3/2_)5*p*	^2^[5/2]	3
49000 S	11177.5227	0.0014	8944.0770	0.0011	2*p*^5^(^2^P^o^_3/2_)3*p*	^2^[5/2]	3	—	2*p*^5^(^2^P^o^_3/2_)4*s*	^2^[3/2]^o^	2
65	11292.9647	0.0015	8852.6466	0.0012	2*p*^5^(^2^P^o^_3/2_)4*s*	^2^[3/2]^o^	1	—	2*p*^5^(^2^P^o^_3/2_)5*p*	^2^[3/2]	2
46	11298.4416	0.0016	8848.3553	0.0013	2*p*^5^(^2^P^o^_3/2_)4*s*	^2^[3/2]^o^	2	—	2*p*^5^(^2^P^o^_3/2_)5*p*	^2^[1/2]	1
140	11303.8878	0.0014	8844.0922	0.0011	2*p*^5^(^2^P^o^_1/2_)4*s*	^2^[1/2]^o^	1	—	2*p*^5^(^2^P^o^_1/2_)5*p*	^2^[3/2]	2
100	11304.5457	0.0014	8843.5775	0.0011	2*p*^5^(^2^P^o^_3/2_)4*s*	^2^[3/2]^o^	1	—	2*p*^5^(^2^P^o^_3/2_)5*p*	^2^[3/2]	1
41	11329.6259	0.0019	8824.0007	0.0015	2*p*^5^(^2^P^o^_1/2_)4*s*	^2^[1/2]^o^	1	—	2*p*^5^(^2^P^o^_1/2_)5*p*	^2^[1/2]	1
37	11333.6873	0.0018	8820.8386	0.0014	2*p*^5^(^2^P^o^_1/2_)4*s*	^2^[1/2]^o^	1	—	2*p*^5^(^2^P^o^_1/2_)5*p*	^2^[3/2]	1
110	11366.6716	0.0014	8795.2420	0.0011	2*p*^5^(^2^P^o^_3/2_)4*s*	^2^[3/2]^o^	1	—	2*p*^5^(^2^P^o^_3/2_)5*p*	^2^[5/2]	2
15000 S	11390.4330	0.0014	8776.8944	0.0011	2*p*^5^(^2^P^o^_3/2_)3*p*	^2^[5/2]	2	—	2*p*^5^(^2^P^o^_3/2_)4*s*	^2^[3/2]^o^	2
8800 S	11409.1336	0.0014	8762.5083	0.0011	2*p*^5^(^2^P^o^_1/2_)3*p*	^2^[3/2]	1	—	2*p*^5^(^2^P^o^_1/2_)4*s*	^2^[1/2]^o^	1
33000 S	11522.7450	0.0014	8676.1124	0.0010	2*p*^5^(^2^P^o^_1/2_)3*p*	^2^[3/2]	2	—	2*p*^5^(^2^P^o^_1/2_)4*s*	^2^[1/2]^o^	1
17000 S	11525.0203	0.0014	8674.3995	0.0011	2*p*^5^(^2^P^o^_3/2_)3*p*	^2^[3/2]	1	—	2*p*^5^(^2^P^o^_3/2_)4*s*	^2^[3/2]^o^	1
9100 S	11536.3446	0.0014	8665.8846	0.0010	2*p*^5^(^2^P^o^_1/2_)3*p*	^2^[1/2]	0	—	2*p*^5^(^2^P^o^_3/2_)3*d*	^2^[3/2]^o^	1
2600 S	11601.5369	0.0014	8617.1887	0.0010	2*p*^5^(^2^P^o^_3/2_)3*p*	^2^[1/2]	0	—	2*p*^5^(^2^P^o^_1/2_)4*s*	^2^[1/2]^o^	1
13000 S	11614.0808	0.0014	8607.8816	0.0010	2*p*^5^(^2^P^o^_1/2_)3*p*	^2^[3/2]	1	—	2*p*^5^(^2^P^o^_1/2_)4*s*	^2^[1/2]^o^	0
2800 S	11688.0028	0.0014	8553.4403	0.0010	2*p*^5^(^2^P^o^_1/2_)3*p*	^2^[1/2]	0	—	2*p*^5^(^2^P^o^_3/2_)3*d*	^2^[1/2]^o^	1
15000 S	11766.7930	0.0014	8496.1667	0.0010	2*p*^5^(^2^P^o^_1/2_)3*p*	^2^[1/2]	1	—	2*p*^5^(^2^P^o^_1/2_)4*s*	^2^[1/2]^o^	1
13000 S	11789.0444	0.0014	8480.1306	0.0010	2*p*^5^(^2^P^o^_3/2_)3*p*	^2^[3/2]	2	—	2*p*^5^(^2^P^o^_3/2_)4*s*	^2^[3/2]^o^	1
3200 S	11789.8894	0.0014	8479.5228	0.0010	2*p*^5^(^2^P^o^_3/2_)3*p*	^2^[3/2]	1	—	2*p*^5^(^2^P^o^_3/2_)4*s*	^2^[3/2]^o^	2
20	11979.781	0.003	8345.1142	0.0019	2*p*^5^(^2^P^o^_3/2_)4*p*	^2^[1/2]	1	—	2*p*^5^(^2^P^o^_3/2_)6*d*	^2^[3/2]^o^	2
7400 S	11984.9139	0.0014	8341.5401	0.0010	2*p*^5^(^2^P^o^1/2)3*p*	^2^[1/2]	1	—	2*p*^5^(^2^P^o^_1/2_)4*s*	^2^[1/2]^o^	0
18	11996.569	0.002	8333.4360	0.0016	2*p*^5^(^2^P^o^_3/2_)4*p*	^2^[1/2]	1	—	2*p*^5^(^2^P^o^_3/2_)6*d*	^2^[1/2]^o^	1
5	11997.813	0.005	8332.572	0.003	2*p*^5^(^2^P^o^_1/2_)4*s*	^2^[1/2]^o^	1	—	2*p*^5^(^2^P^o^_3/2_)5*p*	^2^[1/2]	0
23000 S	12066.3341	0.0014	8285.2538	0.0010	2*p*^5^(^2^P^o^_3/2_)3*p*	^2^[3/2]	2	—	2*p*^5^(^2^P^o^_3/2_)4*s*	^2^[3/2]^o^	2
3	12388.983	0.005	8069.480	0.003	2*p*^5^(^2^P^o^_1/2_)4*s*	^2^[1/2]^o^	0	—	2*p*^5^(^2^P^o^_3/2_)5*p*	^2^[1/2]	1
4	12408.769	0.005	8056.613	0.003	2*p*^5^(^2^P^o^_1/2_)4*s*	^2^[1/2]^o^	1	—	2*p*^5^(^2^P^o^_3/2_)5*p*	^2^[5/2]	2
18	12430.505	0.003	8042.5253	0.0016	2*p*^5^(^2^P^o^_3/2_)4*p*	^2^[5/2]	3	—	2*p*^5^(^2^P^o^_3/2_)6*d*	^2^[5/2]^o^	3
75	12453.3684	0.0014	8027.7596	0.0009	2*p*^5^(^2^P^o^_3/2_)4*p*	^2^[5/2]	3	—	2*p*^5^(^2^P^o^_3/2_)6*d*	^2^[7/2]^o^	4
4300 S	12459.3903	0.0016	8023.8796	0.0010	2*p*^5^(^2^P^o^_1/2_)3*p*	^2^[3/2]	1	—	2*p*^5^(^2^P^o^_3/2_)4*s*	^2^[3/2]^o^	1
160	12464.1163	0.0014	8020.8372	0.0009	2*p*^5^(^2^P^o^_3/2_)3*d*	^2^[1/2]^o^	0	—	2*p*^5^(^2^P^o^_3/2_)5*f*	^2^[3/2]	1
23 *	12473.468	0.003	8014.824	0.002	2*p*^5^(^2^P^o^_3/2_)4*p*	^2^[1/2]	1	—	2*p*^5^(^2^P^o^_3/2_)7*s*	^2^[3/2]^o^	2
23 *	12473.468	0.003	8014.824	0.002	2*p*^5^(^2^P^o^_3/2_)3*d*	^2^[1/2]^o^	1	—	2*p*^5^(^2^P^o^_3/2_)5*f*	^2^[5/2]	2
450 *	12486.7315	0.0014	8006.3104	0.0009	2*p*^5^(^2^P^o^_3/2_)3*d*	^2^[1/2]^o^	1	—	2*p*^5^(^2^P^o^_3/2_)5*f*	^2^[3/2]	2
450 *	12486.7315	0.0014	8006.3104	0.0009	2*p*^5^(^2^P^o^_3/2_)3*d*	^2^[1/2]^o^	1	—	2*p*^5^(^2^P^o^_3/2_)5*f*	^2^[3/2]	1
21	12520.2343	0.0018	7984.8864	0.0011	2*p*^5^(^2^P^o^_1/2_)4*p*	^2^[3/2]	1	—	2*p*^5^(^2^P^o^_1/2_)6*d*	^2^[5/2]^o^	2
15	12537.742	0.003	7973.7361	0.0016	2*p*^5^(^2^P^o^_3/2_)4*p*	^2^[5/2]	2	—	2*p*^5^(^2^P^o^_3/2_)6*d*	^2^[5/2]^o^	2
48	12559.7621	0.0015	7959.7566	0.0009	2*p*^5^(^2^P^o^_3/2_)4*p*	^2^[5/2]	2	—	2*p*^5^(^2^P^o^_3/2_)6*d*	^2^[7/2]^o^	3
130	12571.0054	0.0016	7952.6375	0.0010	2*p*^5^(^2^P^o^_3/2_)3*d*	^2^[7/2]^o^	4	—	2*p*^5^(^2^P^o^_3/2_)5*f*	^2^[7/2]	4
96 *	12573.8231	0.0015	7950.8554	0.0009	2*p*^5^(^2^P^o^_3/2_)3*d*	^2^[7/2]^o^	3	—	2*p*^5^(^2^P^o^_3/2_)5*f*	^2^[7/2]	4
96 *	12573.8231	0.0015	7950.8554	0.0009	2*p*^5^(^2^P^o^_3/2_)3*d*	^2^[7/2]^o^	3	—	2*p*^5^(^2^P^o^_3/2_)5*f*	^2^[7/2]	3
8	12577.349	0.005	7948.627	0.003	2*p*^5^(^2^P^o^_3/2_)3*d*	^2^[7/2]^o^	4	—	2*p*^5^(^2^P^o^_3/2_)5*f*	^2^[5/2]	3
6	12580.144	0.004	7946.861	0.002	2*p*^5^(^2^P^o^_3/2_)3*d*	^2^[7/2]^o^	3	—	2*p*^5^(^2^P^o^_3/2_)5*f*	^2^[5/2]	2
1200	12584.6021	0.0014	7944.0453	0.0009	2*p*^5^(^2^P^o^_3/2_)3*d*	^2^[7/2]^o^	4	—	2*p*^5^(^2^P^o^_3/2_)5*f*	^2^[9/2]	5
850	12587.4256	0.0014	7942.2634	0.0009	2*p*^5^(^2^P^o^_3/2_)3*d*	^2^[7/2]^o^	3	—	2*p*^5^(^2^P^o^_3/2_)5*f*	^2^[9/2]	4
1600 S	12595.0049	0.0014	7937.4840	0.0009	2*p*^5^(^2^P^o^_1/2_)3*p*	^2^[3/2]	2	—	2*p*^5^(^2^P^o^_3/2_)4*s*	^2^[3/2]^o^	1
32	12600.7778	0.0019	7933.8475	0.0012	2*p*^5^(^2^P^o^_1/2_)4*p*	^2^[3/2]	2	—	2*p*^5^(^2^P^o^_1/2_)6*d*	^2^[5/2]^o^	3
18	12603.3179	0.0020	7932.2485	0.0013	2*p*^5^(^2^P^o^_1/2_)4*p*	^2^[1/2]	1	—	2*p*^5^(^2^P^o^_1/2_)6*d*	^2^[3/2]^o^	2
550	12604.1773	0.0014	7931.7077	0.0009	2*p*^5^(^2^P^o^_3/2_)3*d*	^2^[3/2]^o^	2	—	2*p*^5^(^2^P^o^_3/2_)5*f*	^2^[5/2]	3
150	12617.6692	0.0014	7923.2264	0.0009	2*p*^5^(^2^P^o^_3/2_)3*d*	^2^[3/2]^o^	2	—	2*p*^5^(^2^P^o^_3/2_)5*f*	^2^[3/2]	2
6	12631.024	0.005	7914.849	0.003	2*p*^5^(^2^P^o^_1/2_)4*s*	^2^[1/2]^o^	1	—	2*p*^5^(^2^P^o^_3/2_)5*p*	^2^[1/2]	1
500	12640.3174	0.0014	7909.0301	0.0009	2*p*^5^(^2^P^o^_1/2_)3*d*	^2^[5/2]^o^	2	—	2*p*^5^(^2^P^o^_1/2_)5*f*	^2^[7/2]	3
710	12642.7394	0.0014	7907.5149	0.0009	2*p*^5^(^2^P^o^_1/2_)3*d*	^2^[5/2]^o^	3	—	2*p*^5^(^2^P^o^_1/2_)5*f*	^2^[7/2]	4
330	12650.9824	0.0014	7902.3626	0.0009	2*p*^5^(^2^P^o^_3/2_)3*d*	^2^[3/2]^o^	1	—	2*p*^5^(^2^P^o^_3/2_)5*f*	^2^[5/2]	2
470	12658.3460	0.0014	7897.7657	0.0009	2*p*^5^(^2^P^o^_1/2_)3*d*	^2^[3/2]^o^	2	—	2*p*^5^(^2^P^o^_1/2_)5*f*	^2^[5/2]	3
98 *	12664.6063	0.0014	7893.8617	0.0009	2*p*^5^(^2^P^o^_3/2_)3*d*	^2^[3/2]^o^	1	—	2*p*^5^(^2^P^o^_3/2_)5*f*	^2^[3/2]	2
98 *	12664.6063	0.0014	7893.8617	0.0009	2*p*^5^(^2^P^o^_3/2_)3*d*	^2^[3/2]^o^	1	—	2*p*^5^(^2^P^o^_3/2_)5*f*	^2^[3/2]	1
300	12683.5329	0.0014	7882.0824	0.0009	2*p*^5^(^2^P^o^_1/2_)3*d*	^2^[3/2]^o^	1	—	2*p*^5^(^2^P^o^_1/2_)5*f*	^2^[5/2]	2
6500 S	12689.2032	0.0014	7878.5602	0.0009	2*p*^5^(^2^P^o^_3/2_)3*p*	^2^[1/2]	0	—	2*p*^5^(^2^P^o^_3/2_)4*s*	^2^[3/2]^o^	1
23	12718.797	0.002	7860.2284	0.0014	2*p*^5^(^2^P^o^_3/2_)4*p*	^2^[3/2]	1	—	2*p*^5^(^2^P^o^_3/2_)6*d*	^2^[5/2]^o^	2
11	12726.785	0.004	7855.295	0.002	2*p*^5^(^2^P^o^_3/2_)4*p*	^2^[3/2]	1	—	2*p*^5^(^2^P^o^_3/2_)6*d*	^2^[3/2]^o^	1
540	12746.2264	0.0015	7843.3137	0.0009	2*p*^5^(^2^P^o^_3/2_)3*d*	^2^[5/2]^o^	2	—	2*p*^5^(^2^P^o^_3/2_)5*f*	^2^[7/2]	3
710	12749.1248	0.0015	7841.5306	0.0009	2*p*^5^(^2^P^o^_3/2_)3*d*	^2^[5/2]^o^	3	—	2*p*^5^(^2^P^o^_3/2_)5*f*	^2^[7/2]	4
120 *	12752.7222	0.0014	7839.3186	0.0009	2*p*^5^(^2^P^o^_3/2_)3*d*	^2^[5/2]^o^	2	—	2*p*^5^(^2^P^o^_3/2_)5*f*	^2^[5/2]	2
120 *	12752.7222	0.0014	7839.3186	0.0009	2*p*^5^(^2^P^o^_3/2_)3*d*	^2^[5/2]^o^	2	—	2*p*^5^(^2^P^o^_3/2_)5*f*	^2^[5/2]	3
170	12755.6507	0.0014	7837.5188	0.0009	2*p*^5^(^2^P^o^_3/2_)3*d*	^2^[5/2]^o^	3	—	2*p*^5^(^2^P^o^_3/2_)5*f*	^2^[5/2]	3
37	12759.9494	0.0016	7834.8784	0.0010	2*p*^5^(^2^P^o^_3/2_)4*p*	^2^[3/2]	2	—	2*p*^5^(^2^P^o^_3/2_)6*d*	^2^[5/2]^o^	3
6 *	12766.582	0.003	7830.808	0.002	2*p*^5^(^2^P^o^_3/2_)3*d*	^2^[5/2]^o^	2	—	2*p*^5^(^2^P^o^_3/2_)5*f*	^2^[3/2]	2
6 *	12766.582	0.003	7830.808	0.002	2*p*^5^(^2^P^o^_3/2_)3*d*	^2^[5/2]^o^	2	—	2*p*^5^(^2^P^o^_3/2_)5*f*	^2^[3/2]	1
1600 S	12769.5250	0.0014	7829.0032	0.0009	2*p*^5^(^2^P^o^_1/2_)3*p*	^2^[3/2]	1	—	2*p*^5^(^2^P^o^_3/2_)4*s*	^2^[3/2]^o^	2
16	12776.652	0.003	7824.6359	0.0020	2*p*^5^(^2^P^o^_3/2_)4*p*	^2^[3/2]	2	—	2*p*^5^(^2^P^o^_3/2_)6*d*	^2^[3/2]^o^	2
5	12853.034	0.004	7778.136	0.003	2*p*^5^(^2^P^o^_3/2_)4*p*	^2^[1/2]	1	—	2*p*^5^(^2^P^o^_1/2_)5*d*	^2^[3/2]^o^	1
7	12864.091	0.003	7771.451	0.002	2*p*^5^(^2^P^o^_3/2_)4*p*	^2^[1/2]	1	—	2*p*^5^(^2^P^o^_1/2_)5*d*	^2^[5/2]^o^	2
11	12864.730	0.002	7771.0649	0.0013	2*p*^5^(^2^P^o^_3/2_)4*p*	^2^[1/2]	1	—	2*p*^5^(^2^P^o^_1/2_)5*d*	^2^[3/2]^o^	2
14	12887.1630	0.0017	7757.5378	0.0010	2*p*^5^(^2^P^o^_1/2_)3*p*	^2^[1/2]	1	—	2*p*^5^(^2^P^o^_3/2_)4*s*	^2^[3/2]^o^	1
8400 S	12912.0141	0.0014	7742.6073	0.0008	2*p*^5^(^2^P^o^_1/2_)3*p*	^2^[3/2]	2	—	2*p*^5^(^2^P^o^_3/2_)4*s*	^2^[3/2]^o^	2
25	12980.0736	0.0015	7702.0099	0.0009	2*p*^5^(^2^P^o^_3/2_)4*p*	^2^[5/2]	3	—	2*p*^5^(^2^P^o^_3/2_)7*s*	^2^[3/2]^o^	2
12	13054.788	0.003	7657.9304	0.0017	2*p*^5^(^2^P^o^_3/2_)4*p*	^2^[5/2]	2	—	2*p*^5^(^2^P^o^_3/2_)7*s*	^2^[3/2]^o^	1
6	13058.815	0.003	7655.5690	0.0016	2*p*^5^(^2^P^o^_1/2_)4*p*	^2^[3/2]	1	—	2*p*^5^(^2^P^o^_1/2_)7*s*	^2^[1/2]^o^	0
6	13096.396	0.004	7633.601	0.002	2*p*^5^(^2^P^o^_3/2_)4*p*	^2^[5/2]	2	—	2*p*^5^(^2^P^o^_3/2_)7*s*	^2^[3/2]^o^	2
6	13126.171	0.005	7616.285	0.003	2*p*^5^(^2^P^o^_1/2_)4*p*	^2^[1/2]	1	—	2*p*^5^(^2^P^o^_1/2_)7*s*	^2^[1/2]^o^	1
12	13127.681	0.002	7615.4087	0.0014	2*p*^5^(^2^P^o^_1/2_)4*p*	^2^[3/2]	2	—	2*p*^5^(^2^P^o^_1/2_)7*s*	^2^[1/2]^o^	1
5	13145.446	0.005	7605.117	0.003	2*p*^5^(^2^P^o^_1/2_)4*p*	^2^[1/2]	1	—	2*p*^5^(^2^P^o^_1/2_)7*s*	^2^[1/2]^o^	0
4500 S	13219.2426	0.0014	7562.6616	0.0008	2*p*^5^(^2^P^o^_1/2_)3*p*	^2^[1/2]	1	—	2*p*^5^(^2^P^o^_3/2_)4*s*	^2^[3/2]^o^	2
8	13251.199	0.002	7544.4239	0.0013	2*p*^5^(^2^P^o^_3/2_)4*p*	^2^[3/2]	1	—	2*p*^5^(^2^P^o^_3/2_)7*s*	^2^[3/2]^o^	1
6	13296.547	0.004	7518.6934	0.0020	2*p*^5^(^2^P^o^_3/2_)4*p*	^2^[3/2]	2	—	2*p*^5^(^2^P^o^_3/2_)7*s*	^2^[3/2]^o^	1
10	13339.714	0.003	7494.3634	0.0017	2*p*^5^(^2^P^o^_3/2_)4*p*	^2^[3/2]	2	—	2*p*^5^(^2^P^o^_3/2_)7*s*	^2^[3/2]^o^	2
7	13389.290	0.005	7466.614	0.003	2*p*^5^(^2^P^o^_3/2_)4*p*	^2^[1/2]	0	—	2*p*^5^(^2^P^o^_3/2_)6*d*	^2^[3/2]^o^	1
5	13527.073	0.004	7390.562	0.002	2*p*^5^(^2^P^o^_3/2_)4*p*	^2^[5/2]	2	—	2*p*^5^(^2^P^o^_1/2_)5*d*	^2^[5/2]^o^	3
5	13585.088	0.007	7359.000	0.003	2*p*^5^(^2^P^o^_1/2_)4*p*	^2^[1/2]	0	—	2*p*^5^(^2^P^o^_1/2_)6*d*	^2^[3/2]^o^	1
4	13738.735	0.004	7276.701	0.002	2*p*^5^(^2^P^o^_3/2_)4*p*	^2^[3/2]	1	—	2*p*^5^(^2^P^o^_1/2_)5*d*	^2^[5/2]^o^	2
9	13866.305	0.002	7209.7555	0.0011	2*p*^5^(^2^P^o^_3/2_)4*p*	^2^[1/2]	1	—	2*p*^5^(^2^P^o^_1/2_)6*s*	^2^[1/2]^o^	1
5	13908.173	0.003	7188.0517	0.0015	2*p*^5^(^2^P^o^_3/2_)4*p*	^2^[1/2]	1	—	2*p*^5^(^2^P^o^_1/2_)6*s*	^2^[1/2]^o^	0
3	13970.972	0.005	7155.742	0.002	2*p*^5^(^2^P^o^_3/2_)4*p*	^2^[1/2]	0	—	2*p*^5^(^2^P^o^_3/2_)7*s*	^2^[3/2]^o^	1
4	14012.921	0.005	7134.320	0.002	2*p*^5^(^2^P^o^_1/2_)3*d*	^2^[5/2]^o^	2	—	2*p*^5^(^2^P^o^_3/2_)5*f*	^2^[7/2]	3
6	14015.900	0.006	7132.804	0.003	2*p*^5^(^2^P^o^_1/2_)3*d*	^2^[5/2]^o^	3	—	2*p*^5^(^2^P^o^_3/2_)5*f*	^2^[7/2]	4
9	14043.107	0.003	7118.9849	0.0014	2*p*^5^(^2^P^o^_1/2_)3*d*	^2^[3/2]^o^	2	—	2*p*^5^(^2^P^o^_3/2_)5*f*	^2^[5/2]	3
5	14074.110	0.004	7103.303	0.002	2*p*^5^(^2^P^o^_1/2_)3*d*	^2^[3/2]^o^	1	—	2*p*^5^(^2^P^o^_3/2_)5*f*	^2^[5/2]	2
7	14283.603	0.011	6999.122	0.005	2*p*^5^(^2^P^o^_3/2_)4*p*	^2^[1/2]	1	—	2*p*^5^(^2^P^o^_3/2_)5*d*	^2^[3/2]^o^	1
130	14300.8338	0.0016	6990.6883	0.0008	2*p*^5^(^2^P^o^_3/2_)4*p*	^2^[1/2]	1	—	2*p*^5^(^2^P^o^_3/2_)5*d*	^2^[3/2]^o^	2
120	14342.1609	0.0016	6970.5446	0.0008	2*p*^5^(^2^P^o^_3/2_)4*p*	^2^[1/2]	1	—	2*p*^5^(^2^P^o^_3/2_)5*d*	^2^[1/2]^o^	1
51	14353.3494	0.0016	6965.1110	0.0008	2*p*^5^(^2^P^o^_3/2_)4*p*	^2^[1/2]	1	—	2*p*^5^(^2^P^o^_3/2_)5*d*	^2^[1/2]^o^	0
5	14384.113	0.005	6950.215	0.002	2*p*^5^(^2^P^o^_3/2_)3*d*	^2^[3/2]^o^	1	—	2*p*^5^(^2^P^o^_1/2_)5*p*	^2^[1/2]	0
18	14499.9217	0.0018	6894.7044	0.0009	2*p*^5^(^2^P^o^_3/2_)4*p*	^2^[1/2]	0	—	2*p*^5^(^2^P^o^_1/2_)5*d*	^2^[3/2]^o^	1
110	14929.8061	0.0017	6696.1806	0.0008	2*p*^5^(^2^P^o^_3/2_)4*p*	^2^[5/2]	3	—	2*p*^5^(^2^P^o^_3/2_)5*d*	^2^[5/2]^o^	3
7	14931.183	0.003	6695.5633	0.0015	2*p*^5^(^2^P^o^_3/2_)4*p*	^2^[5/2]	3	—	2*p*^5^(^2^P^o^_3/2_)5*d*	^2^[5/2]^o^	2
15	14970.774	0.002	6677.8563	0.0009	2*p*^5^(^2^P^o^_3/2_)4*p*	^2^[5/2]	3	—	2*p*^5^(^2^P^o^_3/2_)5*d*	^2^[3/2]^o^	2
22	14984.854	0.002	6671.5819	0.0010	2*p*^5^(^2^P^o^_3/2_)4*p*	^2^[5/2]	3	—	2*p*^5^(^2^P^o^_3/2_)5*d*	^2^[7/2]^o^	3
530	14986.3193	0.0016	6670.9294	0.0007	2*p*^5^(^2^P^o^_3/2_)4*p*	^2^[5/2]	3	—	2*p*^5^(^2^P^o^_3/2_)5*d*	^2^[7/2]^o^	4
19	15058.9894	0.0020	6638.7376	0.0009	2*p*^5^(^2^P^o^_1/2_)4*p*	^2^[3/2]	1	—	2*p*^5^(^2^P^o^_1/2_)5*d*	^2^[3/2]^o^	1
140	15074.1688	0.0017	6632.0525	0.0007	2*p*^5^(^2^P^o^_1/2_)4*p*	^2^[3/2]	1	—	2*p*^5^(^2^P^o^_1/2_)5*d*	^2^[5/2]^o^	2
48	15075.0431	0.0017	6631.6679	0.0007	2*p*^5^(^2^P^o^_1/2_)4*p*	^2^[3/2]	1	—	2*p*^5^(^2^P^o^_1/2_)5*d*	^2^[3/2]^o^	2
4	15083.912	0.009	6627.769	0.004	2*p*^5^(^2^P^o^_3/2_)4*p*	^2^[5/2]	2	—	2*p*^5^(^2^P^o^_3/2_)5*d*	^2^[5/2]^o^	3
94	15085.3103	0.0017	6627.1543	0.0007	2*p*^5^(^2^P^o^_3/2_)4*p*	^2^[5/2]	2	—	2*p*^5^(^2^P^o^_3/2_)5*d*	^2^[5/2]^o^	2
13	15106.454	0.002	6617.8785	0.0010	2*p*^5^(^2^P^o^_3/2_)4*p*	^2^[5/2]	2	—	2*p*^5^(^2^P^o^_3/2_)5*d*	^2^[3/2]^o^	1
5	15125.728	0.005	6609.446	0.002	2*p*^5^(^2^P^o^_3/2_)4*p*	^2^[5/2]	2	—	2*p*^5^(^2^P^o^_3/2_)5*d*	^2^[3/2]^o^	2
350	15140.0981	0.0017	6603.1725	0.0007	2*p*^5^(^2^P^o^_3/2_)4*p*	^2^[5/2]	2	—	2*p*^5^(^2^P^o^_3/2_)5*d*	^2^[7/2]^o^	3
6	15171.965	0.004	6589.3035	0.0015	2*p*^5^(^2^P^o^_3/2_)4*p*	^2^[5/2]	2	—	2*p*^5^(^2^P^o^_3/2_)5*d*	^2^[1/2]^o^	1
32	15174.3113	0.0019	6588.2845	0.0008	2*p*^5^(^2^P^o^_1/2_)4*p*	^2^[1/2]	1	—	2*p*^5^(^2^P^o^_1/2_)5*d*	^2^[3/2]^o^	1
4	15176.335	0.007	6587.406	0.003	2*p*^5^(^2^P^o^_1/2_)4*p*	^2^[3/2]	2	—	2*p*^5^(^2^P^o^_1/2_)5*d*	^2^[3/2]^o^	1
63	15189.7238	0.0018	6581.5996	0.0008	2*p*^5^(^2^P^o^_1/2_)4*p*	^2^[1/2]	1	—	2*p*^5^(^2^P^o^_1/2_)5*d*	^2^[5/2]^o^	2
99	15190.6122	0.0017	6581.2147	0.0007	2*p*^5^(^2^P^o^_1/2_)4*p*	^2^[1/2]	1	—	2*p*^5^(^2^P^o^_1/2_)5*d*	^2^[3/2]^o^	2
270	15190.9319	0.0017	6581.0762	0.0007	2*p*^5^(^2^P^o^_1/2_)4*p*	^2^[3/2]	2	—	2*p*^5^(^2^P^o^_1/2_)5*d*	^2^[5/2]^o^	3
48	15192.6365	0.0018	6580.3378	0.0008	2*p*^5^(^2^P^o^_1/2_)4*p*	^2^[3/2]	2	—	2*p*^5^(^2^P^o^_1/2_)5*d*	^2^[3/2]^o^	2
5300 S	15230.7144	0.0017	6563.8865	0.0007	2*p*^5^(^2^P^o^_1/2_)3*p*	^2^[1/2]	0	—	2*p*^5^(^2^P^o^_1/2_)4*s*	^2^[1/2]^o^	1
160	15348.1896	0.0017	6513.6465	0.0007	2*p*^5^(^2^P^o^_3/2_)4*p*	^2^[3/2]	1	—	2*p*^5^(^2^P^o^_3/2_)5*d*	^2^[5/2]^o^	2
74	15370.0789	0.0017	6504.3701	0.0007	2*p*^5^(^2^P^o^_3/2_)4*p*	^2^[3/2]	1	—	2*p*^5^(^2^P^o^_3/2_)5*d*	^2^[3/2]^o^	1
17	15390.028	0.002	6495.9391	0.0009	2*p*^5^(^2^P^o^_3/2_)4*p*	^2^[3/2]	1	—	2*p*^5^(^2^P^o^_3/2_)5*d*	^2^[3/2]^o^	2
250	15407.5930	0.0017	6488.5334	0.0007	2*p*^5^(^2^P^o^_3/2_)4*p*	^2^[3/2]	2	—	2*p*^5^(^2^P^o^_3/2_)5*d*	^2^[5/2]^o^	3
6	15409.058	0.006	6487.916	0.002	2*p*^5^(^2^P^o^_3/2_)4*p*	^2^[3/2]	2	—	2*p*^5^(^2^P^o^_3/2_)5*d*	^2^[5/2]^o^	2
4	15431.122	0.007	6478.640	0.003	2*p*^5^(^2^P^o^_3/2_)4*p*	^2^[3/2]	2	—	2*p*^5^(^2^P^o^_3/2_)5*d*	^2^[3/2]^o^	1
11	15450.863	0.003	6470.3623	0.0014	2*p*^5^(^2^P^o^_3/2_)4*p*	^2^[3/2]	1	—	2*p*^5^(^2^P^o^_3/2_)5*d*	^2^[1/2]^o^	0
110	15451.2285	0.0017	6470.2093	0.0007	2*p*^5^(^2^P^o^_3/2_)4*p*	^2^[3/2]	2	—	2*p*^5^(^2^P^o^_3/2_)5*d*	^2^[3/2]^o^	2
25	15466.2267	0.0019	6463.9349	0.0008	2*p*^5^(^2^P^o^_3/2_)4*p*	^2^[3/2]	2	—	2*p*^5^(^2^P^o^_3/2_)5*d*	^2^[7/2]^o^	3
23	15499.484	0.002	6450.0653	0.0009	2*p*^5^(^2^P^o^_3/2_)4*p*	^2^[3/2]	2	—	2*p*^5^(^2^P^o^_3/2_)5*d*	^2^[1/2]^o^	1
13	15500.897	0.003	6449.4773	0.0011	2*p*^5^(^2^P^o^_3/2_)4*p*	^2^[1/2]	1	—	2*p*^5^(^2^P^o^_3/2_)6*s*	^2^[3/2]^o^	1
65	15604.2140	0.0017	6406.7747	0.0007	2*p*^5^(^2^P^o^_3/2_)4*p*	^2^[1/2]	1	—	2*p*^5^(^2^P^o^_3/2_)6*s*	^2^[3/2]^o^	2
4	15761.050	0.004	6343.0219	0.0018	2*p*^5^(^2^P^o^_3/2_)3*d*	^2^[1/2]^o^	1	—	2*p*^5^(^2^P^o^_3/2_)5*p*	^2^[1/2]	0
4	15802.647	0.005	6326.3254	0.0018	2*p*^5^(^2^P^o^_3/2_)4*p*	^2^[1/2]	0	—	2*p*^5^(^2^P^o^_1/2_)6*s*	^2^[1/2]^o^	1
7	15812.181	0.004	6322.5108	0.0015	2*p*^5^(^2^P^o^_3/2_)3*d*	^2^[1/2]^o^	1	—	2*p*^5^(^2^P^o^_1/2_)4*f*	^2^[5/2]	2
130	16022.7694	0.0018	6239.4137	0.0007	2*p*^5^(^2^P^o^_3/2_)3*d*	^2^[3/2]^o^	2	—	2*p*^5^(^2^P^o^_1/2_)4*f*	^2^[5/2]	3
5	16045.498	0.003	6230.5754	0.0013	2*p*^5^(^2^P^o^_3/2_)3*d*	^2^[3/2]^o^	1	—	2*p*^5^(^2^P^o^_3/2_)5*p*	^2^[1/2]	0
47	16098.4859	0.0018	6210.0677	0.0007	2*p*^5^(^2^P^o^_3/2_)3*d*	^2^[3/2]^o^	1	—	2*p*^5^(^2^P^o^_1/2_)4*f*	^2^[5/2]	2
5	16252.672	0.007	6151.154	0.003	2*p*^5^(^2^P^o^_1/2_)3*d*	^2^[3/2]^o^	1	—	2*p*^5^(^2^P^o^_1/2_)5*p*	^2^[1/2]	0
7	16263.592	0.005	6147.0238	0.0019	2*p*^5^(^2^P^o^_3/2_)3*d*	^2^[5/2]^o^	2	—	2*p*^5^(^2^P^o^_1/2_)4*f*	^2^[5/2]	2
44	16264.2476	0.0019	6146.7761	0.0007	2*p*^5^(^2^P^o^_3/2_)3*d*	^2^[5/2]^o^	2	—	2*p*^5^(^2^P^o^_1/2_)4*f*	^2^[7/2]	3
8	16268.353	0.003	6145.2250	0.0009	2*p*^5^(^2^P^o^_3/2_)3*d*	^2^[5/2]^o^	3	—	2*p*^5^(^2^P^o^_1/2_)4*f*	^2^[5/2]	3
63	16268.9559	0.0018	6144.9972	0.0007	2*p*^5^(^2^P^o^_3/2_)3*d*	^2^[5/2]^o^	3	—	2*p*^5^(^2^P^o^_1/2_)4*f*	^2^[7/2]	4
40	16346.9230	0.0019	6115.6885	0.0007	2*p*^5^(^2^P^o^_3/2_)4*p*	^2^[1/2]	0	—	2*p*^5^(^2^P^o^_3/2_)5*d*	^2^[3/2]^o^	1
150	16405.2557	0.0018	6093.9428	0.0007	2*p*^5^(^2^P^o^_3/2_)4*p*	^2^[5/2]	3	—	2*p*^5^(^2^P^o^_3/2_)6*s*	^2^[3/2]^o^	2
15	16423.662	0.003	6087.1132	0.0010	2*p*^5^(^2^P^o^_3/2_)4*p*	^2^[1/2]	0	—	2*p*^5^(^2^P^o^_3/2_)5*d*	^2^[1/2]^o^	1
19	16468.993	0.002	6070.3586	0.0008	2*p*^5^(^2^P^o^_1/2_)4*p*	^2^[3/2]	1	—	2*p*^5^(^2^P^o^_1/2_)6*s*	^2^[1/2]^o^	1
81	16474.7531	0.0018	6068.2360	0.0007	2*p*^5^(^2^P^o^_3/2_)4*p*	^2^[5/2]	2	—	2*p*^5^(^2^P^o^_3/2_)6*s*	^2^[3/2]^o^	1
34	16528.0869	0.0019	6048.6547	0.0007	2*p*^5^(^2^P^o^_1/2_)4*p*	^2^[3/2]	1	—	2*p*^5^(^2^P^o^_1/2_)6*s*	^2^[1/2]^o^	0
34	16591.5089	0.0019	6025.5334	0.0007	2*p*^5^(^2^P^o^_3/2_)4*p*	^2^[5/2]	2	—	2*p*^5^(^2^P^o^_3/2_)6*s*	^2^[3/2]^o^	2
30	16607.020	0.002	6019.9054	0.0008	2*p*^5^(^2^P^o^_1/2_)4*p*	^2^[1/2]	1	—	2*p*^5^(^2^P^o^_1/2_)6*s*	^2^[1/2]^o^	1
89	16609.4386	0.0018	6019.0289	0.0007	2*p*^5^(^2^P^o^_1/2_)4*p*	^2^[3/2]	2	—	2*p*^5^(^2^P^o^_1/2_)6*s*	^2^[1/2]^o^	1
35	16634.0497	0.0019	6010.1234	0.0007	2*p*^5^(^2^P^o^_1/2_)4*p*	^2^[1/2]	0	—	2*p*^5^(^2^P^o^_1/2_)5*d*	^2^[3/2]^o^	1
16	16667.111	0.002	5998.2016	0.0008	2*p*^5^(^2^P^o^_1/2_)4*p*	^2^[1/2]	1	—	2*p*^5^(^2^P^o^_1/2_)6*s*	^2^[1/2]^o^	0
54	16788.7921	0.0019	5954.7281	0.0007	2*p*^5^(^2^P^o^_3/2_)4*p*	^2^[3/2]	1	—	2*p*^5^(^2^P^o^_3/2_)6*s*	^2^[3/2]^o^	1
37	16861.6497	0.0020	5928.9983	0.0007	2*p*^5^(^2^P^o^_3/2_)4*p*	^2^[3/2]	2	—	2*p*^5^(^2^P^o^_3/2_)6*s*	^2^[3/2]^o^	1
11	16910.058	0.002	5912.0253	0.0008	2*p*^5^(^2^P^o^_3/2_)4*p*	^2^[3/2]	1	—	2*p*^5^(^2^P^o^_3/2_)6*s*	^2^[3/2]^o^	2
67	16983.9743	0.0019	5886.2956	0.0007	2*p*^5^(^2^P^o^_3/2_)4*p*	^2^[3/2]	2	—	2*p*^5^(^2^P^o^_3/2_)6*s*	^2^[3/2]^o^	2
5	17112.136	0.006	5842.2101	0.0019	2*p*^5^(^2^P^o^_3/2_)3*d*	^2^[3/2]^o^	2	—	2*p*^5^(^2^P^o^_3/2_)5*p*	^2^[1/2]	1
1800 S	17161.9348	0.0019	5825.2578	0.0006	2*p*^5^(^2^P^o^_1/2_)3*p*	^2^[1/2]	0	—	2*p*^5^(^2^P^o^_3/2_)4*s*	^2^[3/2]^o^	1
7	17234.185	0.005	5800.8367	0.0018	2*p*^5^(^2^P^o^_1/2_)4*p*	^2^[1/2]	1	—	2*p*^5^(^2^P^o^_3/2_)5*d*	^2^[3/2]^o^	2
7	17294.240	0.006	5780.693	0.002	2*p*^5^(^2^P^o^_1/2_)4*p*	^2^[1/2]	1	—	2*p*^5^(^2^P^o^_3/2_)5*d*	^2^[1/2]^o^	1
4	17310.510	0.006	5775.2599	0.0019	2*p*^5^(^2^P^o^_1/2_)4*p*	^2^[1/2]	1	—	2*p*^5^(^2^P^o^_3/2_)5*d*	^2^[1/2]^o^	0
13	17961.168	0.002	5566.0468	0.0007	2*p*^5^(^2^P^o^_3/2_)4*p*	^2^[1/2]	0	—	2*p*^5^(^2^P^o^_3/2_)6*s*	^2^[3/2]^o^	1
160	18029.6473	0.0020	5544.9060	0.0006	2*p*^5^(^2^P^o^_3/2_)3*d*	^2^[1/2]^o^	1	—	2*p*^5^(^2^P^o^_3/2_)4*f*	^2^[5/2]	2
1900	18035.8121	0.0020	5543.0107	0.0006	2*p*^5^(^2^P^o^_3/2_)3*d*	^2^[1/2]^o^	0	—	2*p*^5^(^2^P^o^_3/2_)4*f*	^2^[3/2]	1
4500	18083.181	0.003	5528.4909	0.0010	2*p*^5^(^2^P^o^_3/2_)3*d*	^2^[1/2]^o^	1	—	2*p*^5^(^2^P^o^_3/2_)4*f*	^2^[3/2]	2
910	18083.263	0.004	5528.4656	0.0013	2*p*^5^(^2^P^o^_3/2_)3*d*	^2^[1/2]^o^	1	—	2*p*^5^(^2^P^o^_3/2_)4*f*	^2^[3/2]	1
250	18210.3066	0.0020	5489.8966	0.0006	2*p*^5^(^2^P^o^_3/2_)4*s*	^2^[3/2]^o^	1	—	2*p*^5^(^2^P^o^_1/2_)4*p*	^2^[1/2]	0
1600	18221.0868	0.0020	5486.6486	0.0006	2*p*^5^(^2^P^o^_3/2_)3*d*	^2^[7/2]^o^	4	—	2*p*^5^(^2^P^o^_3/2_)4*f*	^2^[7/2]	4
1300	18227.0157	0.0020	5484.8639	0.0006	2*p*^5^(^2^P^o^_3/2_)3*d*	^2^[7/2]^o^	3	—	2*p*^5^(^2^P^o^_3/2_)4*f*	^2^[7/2]	3
87	18247.4481	0.0020	5478.7223	0.0006	2*p*^5^(^2^P^o^_3/2_)3*d*	^2^[7/2]^o^	4	—	2*p*^5^(^2^P^o^_3/2_)4*f*	^2^[5/2]	3
64	18253.3198	0.0020	5476.9599	0.0006	2*p*^5^(^2^P^o^)3*d* 3/2	^2^[7/2]^o^	3	—	2*p*^5^(^2^P^o^)4*f* 3/2	^2^[5/2]	2
14000	18276.6415	0.0020	5469.9711	0.0006	2*p*^5^(^2^P^o^)3*d* 3/2	^2^[7/2]^o^	4	—	2*p*^5^(^2^P^o^)4*f* 3/2	^2^ [9/2]	5
10000	18282.6140	0.0020	5468.1842	0.0006	2*p*^5^(^2^P^o^)3*d* 3/2	^2^[7/2]^o^	3	—	2*p*^5^(^2^P^o^)4*f* 3/2	^2^[9/2]	4
6800	18303.9674	0.0020	5461.8050	0.0006	2*p*^5^(^2^P^o^)3*d* 3/2	^2^[3/2]^o^	2	—	2*p*^5^(^2^P^o^)4*f* 3/2	^2^[5/2]	3
1900	18359.0945	0.0020	5445.4048	0.0006	2*p*^5^(^2^P^o^)3*d* 3/2	^2^[3/2]^o^	2	—	2*p*^5^(^2^P^o^)4*f* 3/2	^2^[3/2]	2
9	18371.441	0.003	5441.7452	0.0009	2*p*^5^(^2^P^o^)4*p* 1/2	^2^[1/2]	0	—	2*p*^5^(^2^P^o^)6*s* 1/2	^2^[1/2]^o^	1
360	18383.9858	0.0020	5438.0319	0.0006	2*p*^5^(^2^P^o^)3*d* 1/2	^2^[5/2]^o^	2	—	2*p*^5^(^2^P^o^)4*f* 1/2	^2^[5/2]	2
6400	18384.8256	0.0020	5437.7835	0.0006	2*p*^5^(^2^P^o^)3*d* 1/2	^2^[5/2]^o^	2	—	2*p*^5^(^2^P^o^)4*f* 1/2	^2^[7/2]	3
480	18389.1674	0.0020	5436.4996	0.0006	2*p*^5^(^2^P^o^)3*d* 1/2	^2^[5/2]^o^	3	—	2*p*^5^(^2^P^o^)4*f* 1/2	^2^[5/2]	3
8600	18389.9366	0.0020	5436.2722	0.0006	2*p*^5^(^2^P^o^)3*d* 1/2	^2^[5/2]^o^	3	—	2*p*^5^(^2^P^o^)4*f* 1/2	^2^[7/2]	4
3900	18402.8356	0.0020	5432.4618	0.0006	2*p*^5^(^2^P^o^)3*d* 3/2	^2^[3/2]^o^	1	—	2*p*^5^(^2^P^o^)4*f* 3/2	^2^[5/2]	2
6300	18422.4016	0.0020	5426.6921	0.0006	2*p*^5^(^2^P^o^)3*d* 1/2	^2^[3/2]^o^	2	—	2*p*^5^(^2^P^o^)4*f* 1/2	^2^[5/2]	3
26	18423.200	0.007	5426.457	0.002	2*p*^5^(^2^P^o^)3*d* 1/2	^2^[3/2]^o^	2	—	2*p*^5^(^2^P^o^)4*f* 1/2	^2^[7/2]	3
1300 *	18458.6404	0.0020	5416.0382	0.0006	2*p*^5^(^2^P^o^)3*d* 3/2	^2^[3/2]^o^	1	—	2*p*^5^(^2^P^o^)4*f* 3/2	^2^[3/2]	2
1300 *	18458.6404	0.0020	5416.0382	0.0006	2*p*^5^(^2^P^o^)3*d* 3/2	^2^[3/2]^o^	1	—	2*p*^5^(^2^P^o^)4*f* 3/2	^2^[3/2]	1
4100	18475.7997	0.0020	5411.0081	0.0006	2*p*^5^(^2^P^o^)3*d* 1/2	^2^[3/2]^o^	1	—	2*p*^5^(^2^P^o^)4*f* 1/2	^2^[5/2]	2
6900	18591.541	0.002	5377.3221	0.0006	2*p*^5^(^2^P^o^)3*d* 3/2	^2^[5/2]^o^	2	—	2*p*^5^(^2^P^o^)4*f* 3/2	^2^[7/2]	3
9500	18597.698	0.002	5375.5419	0.0006	2*p*^5^(^2^P^o^)3*d* 3/2	^2^[5/2]^o^	3	—	2*p*^5^(^2^P^o^)4*f* 3/2	^2^[7/2]	4
1600	18618.908	0.002	5369.4181	0.0006	2*p*^5^(^2^P^o^)3*d* 3/2	^2^[5/2]^o^	2	—	2*p*^5^(^2^P^o^)4*f* 3/2	^2^[5/2]	2
2000	18625.159	0.002	5367.6161	0.0006	2*p*^5^(^2^P^o^)3*d* 3/2	^2^[5/2]^o^	3	—	2*p*^5^(^2^P^o^)4*f* 3/2	^2^[5/2]	3
87	18676.080	0.002	5352.9812	0.0006	2*p*^5^(^2^P^o^)3*d* 3/2	^2^[5/2]^o^	2	—	2*p*^5^(^2^P^o^)4*f* 3/2	^2^[3/2]	1
130	18682.238	0.002	5351.2167	0.0006	2*p*^5^(^2^P^o^)3*d* 3/2	^2^[5/2]^o^	3	—	2*p*^5^(^2^P^o^)4*f* 3/2	^2^[3/2	2
30	18898.826	0.002	5289.8897	0.0006	2*p*^5^(^2^P^o^)4*p* 3/2	^2^[1/2]	1	—	2*p*^5^(^2^P^o^)4*d* 1/2	^2^[3/2]^o^	1
150	18937.552	0.002	5279.0722	0.0006	2*p*^5^(^2^P^o^)4*p* 3/2	^2^[1/2]	1	—	2*p*^5^(^2^P^o^)4*d* 1/2	^2^[3/2]^o^	2
6	18944.644	0.003	5277.0959	0.0009	2*p*^5^(^2^P^o^)4*p* 3/2	^2^[1/2]	1	—	2*p*^5^(^2^P^o^)4*d* 1/2	^2^[5/2]^o^	2
5	19111.191	0.004	5231.1081	0.0011	2*p*^5^(^2^P^o^)4*p* 1/2	^2^[1/2]	0	—	2*p*^5^(^2^P^o^)5*d* 3/2	^2^[3/2]^o^	1
260	19573.750	0.002	5107.4887	0.0006	2*p*^5^(^2^P^o^)4*s* 3/2	^2^[3/2]^o^	2	—	2*p*^5^(^2^P^o^)4*p* 1/2	^2^[3/2]	2
790	19577.110	0.002	5106.6120	0.0006	2*p*^5^(^2^P^o^)4*s* 3/2	^2^[3/2]^o^	2	—	2*p*^5^(^2^P^o^)4*p* 1/2	^2^[1/2]	1
32	19772.462	0.002	5056.1589	0.0006	2*p*^5^(^2^P^o^)4*s* 3/2	^2^[3/2]^o^	2	—	2*p*^5^(^2^P^o^)4*p* 1/2	^2^[3/2]	1
5	19877.309	0.006	5029.4890	0.0016	2*p*^5^(^2^P^o^)3*d* 1/2	^2^[3/2]^o^	2	—	2*p*^5^(^2^P^o^)5*p* 3/2	^2^[1/2]	1
630	20355.771	0.002	4912.6117	0.0005	2*p*^5^(^2^P^o^_3/2_)4*s*	^2^[3/2]^o^	1	—	2*p*^5^(^2^P^o^_1/2_)4*p*	^2^[3/2]	2
43	20359.404	0.002	4911.7351	0.0006	2*p*^5^(^2^P^o^_3/2_)4*s*	^2^[3/2]^o^	1	—	2*p*^5^(^2^P^o^_1/2_)4*p*	^2^[1/2]	1
2	20372.201	0.009	4908.650	0.002	2*p*^5^(^2^P^o^_3/2_)4*p*	^2^[5/2]	2	—	2*p*^5^(^2^P^o^_1/2_)4*d*	^2^[3/2]^o^	1
8	20417.199	0.003	4897.8314	0.0007	2*p*^5^(^2^P^o^_3/2_)4*p*	^2^[5/2]	2	—	2*p*^5^(^2^P^o^_1/2_)4*d*	^2^[3/2]^o^	2
37	20421.587	0.002	4896.7792	0.0006	2*p*^5^(^2^P^o^_3/2_)4*p*	^2^[5/2]	2	—	2*p*^5^(^2^P^o^_1/2_)4*d*	^2^[5/2]^o^	3
3	20425.447	0.009	4895.854	0.002	2*p*^5^(^2^P^o^_3/2_)4*p*	^2^[5/2]	2	—	2*p*^5^(^2^P^o^_1/2_)4*d*	^2^[5/2]^o^	2
11	20854.446	0.003	4795.1406	0.0006	2*p*^5^(^2^P^o^_3/2_)4*p*	^2^[3/2]	1	—	2*p*^5^(^2^P^o^_1/2_)4*d*	^2^[3/2]^o^	1
7	20901.599	0.005	4784.3229	0.0011	2*p*^5^(^2^P^o^_3/2_)4*p*	^2^[3/2]	1	—	2*p*^5^(^2^P^o^_1/2_)4*d*	^2^[3/2]^o^	2
29	20910.236	0.002	4782.3467	0.0005	2*p*^5^(^2^P^o^_3/2_)4*p*	^2^[3/2]	1	—	2*p*^5^(^2^P^o^_1/2_)4*d*	^2^[5/2]^o^	2
2	20966.936	0.005	4769.4140	0.0011	2*p*^5^(^2^P^o^_3/2_)4*p*	^2^[3/2]	2	—	2*p*^5^(^2^P^o^_1/2_)4*d*	^2^[3/2]^o^	1
11	21014.614	0.004	4758.5932	0.0009	2*p*^5^(^2^P^o^_3/2_)4*p*	^2^[3/2]	2	—	2*p*^5^(^2^P^o^_1/2_)4*d*	^2^[3/2]^o^	2
8	21019.261	0.004	4757.5412	0.0009	2*p*^5^(^2^P^o^_3/2_)4*p*	^2^[3/2]	2	—	2*p*^5^(^2^P^o^_1/2_)4*d*	^2^[5/2]^o^	3
3	21023.342	0.007	4756.6177	0.0015	2*p*^5^(^2^P^o^_3/2_)4*p*	^2^[3/2]	2	—	2*p*^5^(^2^P^o^_1/2_)4*d*	^2^[5/2]^o^	2
2700	21047.013	0.002	4751.2680	0.0005	2*p*^5^(^2^P^o^_1/2_)4*s*	^2^[1/2]^o^	1	—	2*p*^5^(^2^P^o^_1/2_)4*p*	^2^[1/2]	0
11 *	21225.899	0.019	4711.226	0.004	2*p*^5^(^2^P^o^_3/2_)4*d*	^2^[1/2]^o^	1	—	2*p*^5^(^2^P^o^_3/2_)7*f*	^2^[3/2]	1
11 *	21225.899	0.019	4711.226	0.004	2*p*^5^(^2^P^o^_3/2_)4*d*	^2^[1/2]^o^	1	—	2*p*^5^(^2^P^o^_3/2_)7*f*	^2^[3/2]	2
26	21331.283	0.015	4687.951	0.003	2*p*^5^(^2^P^o^_3/2_)4*d*	^2^[7/2]^o^	4	—	2*p*^5^(^2^P^o^_3/2_)7*f*	^2^[9/2]	5
20	21336.307	0.021	4686.847	0.005	2*p*^5^(^2^P^o^_3/2_)4*d*	^2^[7/2]^o^	3	—	2*p*^5^(^2^P^o^_3/2_)7*f*	^2^[9/2]	4
15	21374.55	0.06	4678.462	0.013	2*p*^5^(^2^P^o^_3/2_)4*d*	^2^[3/2]^o^	2	—	2*p*^5^(^2^P^o^_3/2_)7*f*	^2^[5/2]	3
10 *	21392.78	0.04	4674.475	0.008	2*p*^5^(^2^P^o^_1/2_)4*d*	^2^[5/2]^o^	2	—	2*p*^5^(^2^P^o^_1/2_)7*f*	^2^[5/2]	3
10 *	21392.78	0.04	4674.475	0.008	2*p*^5^(^2^P^o^_1/2_)4*d*	^2^[5/2]^o^	2	—	2*p*^5^(^2^P^o^_1/2_)7*f*	^2^[7/2]	3
15	21397.04	0.03	4673.544	0.007	2*p*^5^(^2^P^o^_1/2_)4*d*	^2^[5/2]^o^	3	—	2*p*^5^(^2^P^o^_1/2_)7*f*	^2^[7/2]	4
12	21401.81	0.07	4672.503	0.016	2*p*^5^(^2^P^o^_1/2_)4*d*	^2^[3/2]^o^	2	—	2*p*^5^(^2^P^o^_1/2_)7*f*	^2^[5/2]	3
30	21420.939	0.003	4668.3294	0.0006	2*p*^5^(^2^P^o^_1/2_)3*d*	^2^[5/2]^o^	2	—	2*p*^5^(^2^P^o^_3/2_)4*f*	^2^[7/2]	3
46	21427.883	0.003	4666.8167	0.0006	2*p*^5^(^2^P^o^_1/2_)3*d*	^2^[5/2]^o^	3	—	2*p*^5^(^2^P^o^_3/2_)4*f*	^2^[7/2]	4
13	21457.276	0.004	4660.4239	0.0009	2*p*^5^(^2^P^o^_1/2_)3*d*	^2^[5/2]^o^	2	—	2*p*^5^(^2^P^o^_3/2_)4*f*	^2^[5/2]	2
17	21464.337	0.003	4658.8908	0.0007	2*p*^5^(^2^P^o^_1/2_)3*d*	^2^[5/2]^o^	3	—	2*p*^5^(^2^P^o^_3/2_)4*f*	^2^[5/2]	3
90	21509.615	0.002	4649.0836	0.0005	2*p*^5^(^2^P^o^_1/2_)3*d*	^2^[3/2]^o^	2	—	2*p*^5^(^2^P^o^_3/2_)4*f*	^2^[5/2]	3
8	21534.43	0.08	4643.727	0.018	2*p*^5^(^2^P^o^_3/2_)4*d*	^2^[5/2]^o^	2	—	2*p*^5^(^2^P^o^_3/2_)7*f*	^2^[7/2]	3
18	21539.34	0.05	4642.668	0.012	2*p*^5^(^2^P^o^_3/2_)4*d*	^2^[5/2]^o^	3	—	2*p*^5^(^2^P^o^_3/2_)7*f*	^2^[7/2]	4
20 *	21569.002	0.016	4636.283	0.003	2*p*^5^(^2^P^o^_3/2_)4*f*	^2^[3/2]	1	—	2*p*^5^(^2^P^o^_3/2_)7*g*	^2^[5/2]^o^	2
20 *	21569.002	0.016	4636.283	0.003	2*p*^5^(^2^P^o^_3/2_)4*f*	^2^[3/2]	2	—	2*p*^5^(^2^P^o^_3/2_)7*g*	^2^[5/2]^o^	3
34	21582.413	0.003	4633.4022	0.0006	2*p*^5^(^2^P^o^_1/2_)3*d*	^2^[3/2]^o^	1	—	2*p*^5^(^2^P^o^_3/2_)4*f*	^2^[5/2]	2
32	21585.760	0.003	4632.6837	0.0007	2*p*^5^(^2^P^o^_1/2_)3*d*	^2^[3/2]^o^	2	—	2*p*^5^(^2^P^o^_3/2_)4*f*	^2^[3/2]	2
36 *	21602.349	0.014	4629.126	0.003	2*p*^5^(^2^P^o^_3/2_)4*f*	^2^[9/2]	5	—	2*p*^5^(^2^P^o^_3/2_)7*g*	^2^[11/2]^o^	6
36 *	21602.349	0.014	4629.126	0.003	2*p*^5^(^2^P^o^_3/2_)4*f*	^2^[9/2]	4	—	2*p*^5^(^2^P^o^_3/2_)7*g*	^2^[11/2]^o^	5
15 *	21626.61	0.02	4623.933	0.004	2*p*^5^(^2^P^o^_1/2_)4*f*	^2^[7/2]	4	—	2*p*^5^(^2^P^o^_1/2_)7*g*	^2^[9/2]^o^	5
15 *	21626.61	0.02	4623.933	0.004	2*p*^5^(^2^P^o^_1/2_)4*f*	^2^[7/2]	3	—	2*p*^5^(^2^P^o^_1/2_)7*g*	^2^[9/2]^o^	4
12 *	21627.71	0.05	4623.698	0.011	2*p*^5^(^2^P^o^_1/2_)4*f*	^2^[5/2]	2	—	2*p*^5^(^2^P^o^_1/2_)7*g*	^2^[7/2]^o^	3
12 *	21627.71	0.05	4623.698	0.011	2*p*^5^(^2^P^o^_1/2_)4*f*	^2^[5/2]	3	—	2*p*^5^(^2^P^o^_1/2_)7*g*	^2^[7/2]^o^	4
23 *	21638.39	0.07	4621.416	0.016	2*p*^5^(^2^P^o^_3/2_)4*f*	^2^[5/2]	2	—	2*p*^5^(^2^P^o^_3/2_)7*g*	^2^[7/2]^o^	3
23 *	21638.39	0.07	4621.416	0.016	2*p*^5^(^2^P^o^_3/2_)4*f*	^2^[5/2]	3	—	2*p*^5^(^2^P^o^_3/2_)7*g*	^2^[7/2]^o^	4
3 *	21645.61	0.06	4619.875	0.012	2*p*^5^(^2^P^o^_3/2_)4*f*	^2^[5/2]	3	—	2*p*^5^(^2^P^o^_3/2_)7*g*	^2^[5/2]^o^	3
3 *	21645.61	0.06	4619.875	0.012	2*p*^5^(^2^P^o^_3/2_)5*p*	^2^[5/2]	2	—	2*p*^5^(^2^P^o^_3/2_)8*d*	^2^[5/2]^o^	2
3 *	21645.61	0.06	4619.875	0.012	2*p*^5^(^2^P^o^_3/2_)4*f*	^2^[5/2]	2	—	2*p*^5^(^2^P^o^_3/2_)7*g*	^2^[5/2]^o^	3
3 *	21645.61	0.06	4619.875	0.012	2*p*^5^(^2^P^o^_3/2_)4*f*	^2^[5/2]	2	—	2*p*^5^(^2^P^o^_3/2_)7*g*	^2^[5/2]^o^	2
15	21659.251	0.004	4616.9648	0.0009	2*p*^5^(^2^P^o^_1/2_)3*d*	^2^[3/2]^o^	1	—	2*p*^5^(^2^P^o^_3/2_)4*f*	^2^[3/2]	1
23 *	21673.267	0.012	4613.979	0.003	2*p*^5^(^2^P^o^_3/2_)4*f*	^2^[7/2]	3	—	2*p*^5^(^2^P^o^_3/2_)7*g*	^2^[9/2]^o^	4
23 *	21673.267	0.012	4613.979	0.003	2*p*^5^(^2^P^o^_3/2_)4*f*	^2^[7/2]	4	—	2*p*^5^(^2^P^o^_3/2_)7*g*	^2^[9/2]^o^	5
6	21674.67	0.06	4613.681	0.012	2*p*^5^(^2^P^o^_1/2_)5*p*	^2^[1/2]	1	—	2*p*^5^(^2^P^o^_3/2_)12*s*	^2^[3/2]^o^	1
2900	21714.039	0.002	4605.3155	0.0005	2*p*^5^(^2^P^o^_3/2_)4*s*	^2^[3/2]^o^	1	—	2*p*^5^(^2^P^o^_3/2_)4*p*	^2^[1/2]	0
78	22177.292	0.007	4509.1169	0.0014	2*p*^5^(^2^P^o^_3/2_)4*p*	^2^[1/2]	1	—	2*p*^5^(^2^P^o^_3/2_)4*d*	^2^[3/2]^o^	1
1300	22253.432	0.003	4493.6889	0.0005	2*p*^5^(^2^P^o^_3/2_)4*p*	^2^[1/2]	1	—	2*p*^5^(^2^P^o^_3/2_)4*d*	^2^[3/2]^o^	2
1300	22434.265	0.003	4457.4672	0.0005	2*p*^5^(^2^P^o^_3/2_)4*p*	^2^[1/2]	1	—	2*p*^5^(^2^P^o^_3/2_)4*d*	^2^[1/2]^o^	1
540	22472.920	0.003	4449.8001	0.0005	2*p*^5^(^2^P^o^_3/2_)4*p*	^2^[1/2]	1	—	2*p*^5^(^2^P^o^_3/2_)4*d*	^2^[1/2]^o^	0
8500	22536.528	0.003	4437.2408	0.0005	2*p*^5^(^2^P^o^_3/2_)4*s*	^2^[3/2]^o^	2	—	2*p*^5^(^2^P^o^_3/2_)4*p*	^2^[3/2]	2
1300	22667.971	0.003	4411.5108	0.0005	2*p*^5^(^2^P^o^_3/2_)4*s*	^2^[3/2]^o^	2	—	2*p*^5^(^2^P^o^_3/2_)4*p*	^2^[3/2]	1
210	22693.959	0.003	4406.4591	0.0005	2*p*^5^(^2^P^o^_3/2_)4*p*	^2^[1/2]	0	—	2*p*^5^(^2^P^o^_1/2_)4*d*	^2^[3/2]^o^	1
2500	23106.784	0.003	4327.7334	0.0005	2*p*^5^(^2^P^o^_1/2_)4*s*	^2^[1/2]^o^	0	—	2*p*^5^(^2^P^o^_1/2_)4*p*	^2^[1/2]	1
3800	23266.619	0.003	4298.0031	0.0005	2*p*^5^(^2^P^o^_3/2_)4*s*	^2^[3/2]^o^	2	—	2*p*^5^(^2^P^o^_3/2_)4*p*	^2^[5/2]	2
5000	23379.343	0.003	4277.2802	0.0005	2*p*^5^(^2^P^o^_1/2_)4*s*	^2^[1/2]^o^	0	—	2*p*^5^(^2^P^o^_1/2_)4*p*	^2^[3/2]	1
3400	23571.764	0.003	4242.3638	0.0005	2*p*^5^(^2^P^o^_3/2_)4*s*	^2^[3/2]^o^	1	—	2*p*^5^(^2^P^o^_3/2_)4*p*	^2^[3/2]	2
17000	23642.934	0.003	4229.5935	0.0005	2*p*^5^(^2^P^o^_3/2_)4*s*	^2^[3/2]^o^	2	—	2*p*^5^(^2^P^o^_3/2_)4*p*	^2^[5/2]	3
1200	23708.130	0.003	4217.9623	0.0005	2*p*^5^(^2^P^o^_3/2_)4*p*	^2^[5/2]	3	—	2*p*^5^(^2^P^o^_3/2_)4*d*	^2^[5/2]^o^	3
74	23714.099	0.003	4216.9007	0.0005	2*p*^5^(^2^P^o^_3/2_)4*p*	^2^[5/2]	3	—	2*p*^5^(^2^P^o^_3/2_)4*d*	^2^[5/2]^o^	2
5900	23715.599	0.003	4216.6339	0.0005	2*p*^5^(^2^P^o^_3/2_)4*s*	^2^[3/2]^o^	1	—	2*p*^5^(^2^P^o^_3/2_)4*p*	^2^[3/2]	1
170	23918.541	0.003	4180.8571	0.0005	2*p*^5^(^2^P^o^_3/2_)4*p*	^2^[5/2]	3	—	2*p*^5^(^2^P^o^_3/2_)4*d*	^2^[3/2]^o^	2
11000	23957.931	0.003	4173.9831	0.0005	2*p*^5^(^2^P^o^_1/2_)4*s*	^2^[1/2]^o^	1	—	2*p*^5^(^2^P^o^_1/2_)4*p*	^2^[3/2]	2
4600	23962.964	0.003	4173.1065	0.0005	2*p*^5^(^2^P^o^_1/2_)4*s*	^2^[1/2]^o^	1	—	2*p*^5^(^2^P^o^_1/2_)4*p*	^2^[1/2]	1
220	23978.372	0.003	4170.4249	0.0005	2*p*^5^(^2^P^o^_3/2_)4*p*	^2^[5/2]	3	—	2*p*^5^(^2^P^o^_3/2_)4*d*	^2^[7/2]^o^	3
6000	23984.701	0.003	4169.3244	0.0005	2*p*^5^(^2^P^o^_3/2_)4*p*	^2^[5/2]	3	—	2*p*^5^(^2^P^o^_3/2_)4*d*	^2^[7/2]^o^	4
200	24093.528	0.003	4150.4923	0.0005	2*p*^5^(^2^P^o^_1/2_)4*p*	^2^[3/2]	1	—	2*p*^5^(^2^P^o^_1/2_)4*d*	^2^[3/2]^o^	1
46	24098.982	0.003	4149.5529	0.0005	2*p*^5^(^2^P^o^_3/2_)4*p*	^2^[5/2]	2	—	2*p*^5^(^2^P^o^_3/2_)4*d*	^2^[5/2]^o^	3
1100	24105.148	0.003	4148.4914	0.0005	2*p*^5^(^2^P^o^_3/2_)4*p*	^2^[5/2]	2	—	2*p*^5^(^2^P^o^_3/2_)4*d*	^2^[5/2]^o^	2
210	24156.486	0.003	4139.6749	0.0005	2*p*^5^(^2^P^o^_1/2_)4*p*	^2^[3/2]	1	—	2*p*^5^(^2^P^o^_1/2_)4*d*	^2^[3/2]^o^	2
12	24162.547	0.005	4138.6365	0.0008	2*p*^5^(^2^P^o^_3/2_)4*p*	^2^[1/2]	1	—	2*p*^5^(^2^P^o^_1/2_)5*s*	^2^[1/2]^o^	1
2000	24168.025	0.003	4137.6984	0.0005	2*p*^5^(^2^P^o^_1/2_)4*p*	^2^[3/2]	1	—	2*p*^5^(^2^P^o^_1/2_)4*d*	^2^[5/2]^o^	2
140	24225.535	0.003	4127.8759	0.0005	2*p*^5^(^2^P^o^_3/2_)4*p*	^2^[5/2]	2	—	2*p*^5^(^2^P^o^_3/2_)4*d*	^2^[3/2]^o^	1
2800	24256.224	0.003	4122.6533	0.0005	2*p*^5^(^2^P^o^_1/2_)4*s*	^2^[1/2]^o^	1	—	2*p*^5^(^2^P^o^_1/2_)4*p*	^2^[3/2]	1
12	24293.066	0.010	4116.4009	0.0005	2*p*^5^(^2^P^o^_3/2_)5*p*	^2^[5/2]	3	—	2*p*^5^(^2^P^o^_3/2_)7*d*	^2^[7/2]^o^	4
38	24316.420	0.003	4112.4475	0.0005	2*p*^5^(^2^P^o^_3/2_)4*p*	^2^[5/2]	2	—	2*p*^5^(^2^P^o^_3/2_)4*d*	^2^[3/2]^o^	2
7400	24371.661	0.003	4103.1262	0.0005	2*p*^5^(^2^P^o^_3/2_)4*s*	^2^[3/2]^o^	1	—	2*p*^5^(^2^P^o^_3/2_)4*p*	^2^[5/2]	2
3800	24378.260	0.003	4102.0155	0.0005	2*p*^5^(^2^P^o^_3/2_)4*p*	^2^[5/2]	2	—	2*p*^5^(^2^P^o^_3/2_)4*d*	^2^[7/2]^o^	3
360	24390.011	0.003	4100.0391	0.0005	2*p*^5^(^2^P^o^_1/2_)4*p*	^2^[1/2]	1	—	2*p*^5^(^2^P^o^_1/2_)4*d*	^2^[3/2]^o^	1
37	24395.228	0.003	4099.1623	0.0005	2*p*^5^(^2^P^o^_1/2_)4*p*	^2^[3/2]	2	—	2*p*^5^(^2^P^o^_1/2_)4*d*	^2^[3/2]^o^	1
1900	24454.531	0.003	4089.2217	0.0005	2*p*^5^(^2^P^o^_1/2_)4*p*	^2^[1/2]	1	—	2*p*^5^(^2^P^o^_1/2_)4*d*	^2^[3/2]^o^	2
12	24459.078	0.004	4088.4616	0.0007	2*p*^5^(^2^P^o^_3/2_)4*p*	^2^[1/2]	1	—	2*p*^5^(^2^P^o^_1/2_)5*s*	^2^[1/2]^o^	0
240	24459.775	0.003	4088.3450	0.0005	2*p*^5^(^2^P^o^_1/2_)4*p*	^2^[3/2]	2	—	2*p*^5^(^2^P^o^_1/2_)4*d*	^2^[3/2]^o^	2
3300	24466.068	0.003	4087.2934	0.0005	2*p*^5^(^2^P^o^_1/2_)4*p*	^2^[3/2]	2	—	2*p*^5^(^2^P^o^_1/2_)4*d*	^2^[5/2]^o^	3
370	24471.606	0.003	4086.3685	0.0005	2*p*^5^(^2^P^o^_1/2_)4*p*	^2^[3/2]	2	—	2*p*^5^(^2^P^o^_1/2_)4*d*	^2^[5/2]^o^	2
7	24482.800	0.016	4084.500	0.003	2*p*^5^(^2^P^o^_3/2_)5*p*	^2^[5/2]	2	—	2*p*^5^(^2^P^o^_3/2_)7*d*	^2^[7/2]^o^	3
4	24510.36	0.02	4079.908	0.004	2*p*^5^(^2^P^o^_1/2_)5*p*	^2^[3/2]	2	—	2*p*^5^(^2^P^o^_1/2_)7*d*	^2^[5/2]^o^	3
55	24532.498	0.003	4076.2258	0.0005	2*p*^5^(^2^P^o^_3/2_)4*p*	^2^[5/2]	2	—	2*p*^5^(^2^P^o^_3/2_)4*d*	^2^[1/2]^o^	1
9	24606.763	0.003	4063.9234	0.0005	2*p*^5^(^2^P^o^_3/2_)5*s*	^2^[3/2]^o^	1	—	2*p*^5^(^2^P^o^_3/2_)6*p*	^2^[1/2]	0
7	24771.40	0.03	4036.914	0.005	2*p*^5^(^2^P^o^_3/2_)5*p*	^2^[3/2]	2	—	2*p*^5^(^2^P^o^_3/2_)7*d*	^2^[5/2]^o^	3
1700	24783.248	0.003	4034.9836	0.0005	2*p*^5^(^2^P^o^_3/2_)4*p*	^2^[3/2]	1	—	2*p*^5^(^2^P^o^_3/2_)4*d*	^2^[5/2]^o^	2
5	24796.287	0.007	4032.8618	0.0011	2*p*^5^(^2^P^o^_1/2_)5*s*	^2^[1/2]^o^	1	—	2*p*^5^(^2^P^o^_1/2_)6*p*	^2^[1/2]	0
21	24902.752	0.008	4015.6205	0.0012	2*p*^5^(^2^P^o^_3/2_)5*s*	^2^[3/2]^o^	2	—	2*p*^5^(^2^P^o^_3/2_)6*p*	^2^[3/2]	2
780	24910.521	0.003	4014.3681	0.0005	2*p*^5^(^2^P^o^_3/2_)4*p*	^2^[3/2]	1	—	2*p*^5^(^2^P^o^_3/2_)4*d*	^2^[3/2]^o^	1
3	24929.689	0.009	4011.2815	0.0015	2*p*^5^(^2^P^o^_3/2_)5*s*	^2^[3/2]^o^	2	—	2*p*^5^(^2^P^o^_3/2_)6*p*	^2^[3/2]	1
2900	24935.696	0.003	4010.3152	0.0005	2*p*^5^(^2^P^o^_3/2_)4*p*	^2^[3/2]	2	—	2*p*^5^(^2^P^o^_3/2_)4*d*	^2^[5/2]^o^	3
46	24942.298	0.004	4009.2537	0.0006	2*p*^5^(^2^P^o^_3/2_)4*p*	^2^[3/2]	2	—	2*p*^5^(^2^P^o^_3/2_)4*d*	^2^[5/2]^o^	2
170	25006.628	0.003	3998.9398	0.0005	2*p*^5^(^2^P^o^_3/2_)4*p*	^2^[3/2]	1	—	2*p*^5^(^2^P^o^_3/2_)4*d*	^2^[3/2]^o^	2
35	25071.216	0.003	3988.6379	0.0005	2*p*^5^(^2^P^o^_3/2_)4*p*	^2^[3/2]	2	—	2*p*^5^(^2^P^o^_3/2_)4*d*	^2^[3/2]^o^	1
7	25084.899	0.005	3986.4622	0.0007	2*p*^5^(^2^P^o^_3/2_)5*s*	^2^[3/2]^o^	2	—	2*p*^5^(^2^P^o^_3/2_)6*p*	^2^[5/2]	2
1300	25168.567	0.003	3973.2099	0.0005	2*p*^5^(^2^P^o^_3/2_)4*p*	^2^[3/2]	2	—	2*p*^5^(^2^P^o^_3/2_)4*d*	^2^[3/2]^o^	2
3	25176.147	0.008	3972.0136	0.0012	2*p*^5^(^2^P^o^_1/2_)5*s*	^2^[1/2]^o^	0	—	2*p*^5^(^2^P^o^_1/2_)6*p*	^2^[1/2]	1
25	25195.185	0.004	3969.0124	0.0007	2*p*^5^(^2^P^o^_3/2_)5*s*	^2^[3/2]^o^	2	—	2*p*^5^(^2^P^o^_3/2_)6*p*	^2^[5/2]	3
300	25234.824	0.003	3962.7778	0.0005	2*p*^5^(^2^P^o^_3/2_)4*p*	^2^[3/2]	2	—	2*p*^5^(^2^P^o^_3/2_)4*d*	^2^[7/2]^o^	3
100	25284.125	0.003	3955.0508	0.0004	2*p*^5^(^2^P^o^_3/2_)4*p*	^2^[3/2]	1	—	2*p*^5^(^2^P^o^_3/2_)4*d*	^2^[1/2]^o^	0
15	25376.263	0.003	3940.6906	0.0005	2*p*^5^(^2^P^o^_1/2_)5*s*	^2^[1/2]^o^	1	—	2*p*^5^(^2^P^o^_1/2_)6*p*	^2^[3/2]	2
280	25400.128	0.003	3936.9881	0.0005	2*p*^5^(^2^P^o^_3/2_)4*p*	^2^[3/2]	2	—	2*p*^5^(^2^P^o^_3/2_)4*d*	^2^[1/2]^o^	1
7	25438.750	0.004	3931.0107	0.0007	2*p*^5^(^2^P^o^_3/2_)5*s*	^2^[3/2]^o^	1	—	2*p*^5^(^2^P^o^_3/2_)6*p*	^2^[3/2]	2
12	25466.860	0.003	3926.6718	0.0005	2*p*^5^(^2^P^o^_3/2_)5*s*	^2^[3/2]^o^	1	—	2*p*^5^(^2^P^o^_3/2_)6*p*	^2^[3/2]	1
6	25498.245	0.004	3921.8386	0.0006	2*p*^5^(^2^P^o^_1/2_)5*s*	^2^[1/2]^o^	1	—	2*p*^5^(^2^P^o^_1/2_)6*p*	^2^[1/2]	1
6	25510.427	0.006	3919.9657	0.0009	2*p*^5^(^2^P^o^_3/2_)5*s*	^2^[3/2]^o^	2	—	2*p*^5^(^2^P^o^_3/2_)6*p*	^2^[1/2]	1
4600	25531.295	0.003	3916.7618	0.0004	2*p*^5^(^2^P^o^_3/2_)4*s*	^2^[3/2]^o^	2	—	2*p*^5^(^2^P^o^_3/2_)4*p*	^2^[1/2]	1
4	25547.659	0.011	3914.2529	0.0017	2*p*^5^(^2^P^o^_3/2_)5*p*	^2^[5/2]	3	—	2*p*^5^(^2^P^o^_3/2_)8*s*	^2^[3/2]^o^	2
14	25588.111	0.006	3908.0650	0.0010	2*p*^5^(^2^P^o^_3/2_)4*d*	^2^[1/2]^o^	0	—	2*p*^5^(^2^P^o^_3/2_)6*f*	^2^[3/2]	1
7	25628.812	0.004	3901.8586	0.0006	2*p*^5^(^2^P^o^_3/2_)5*s*	^2^[3/2]^o^	1	—	2*p*^5^(^2^P^o^_3/2_)6*p*	^2^[5/2]	2
42	25638.336	0.004	3900.4091	0.0005	2*p*^5^(^2^P^o^_3/2_)4*d*	^2^[1/2]^o^	1	—	2*p*^5^(^2^P^o^_3/2_)6*f*	^2^[3/2]	2
4	25753.06	0.03	3883.033	0.005	2*p*^5^(^2^P^o^_1/2_)5*p*	^2^[3/2]	2	—	2*p*^5^(^2^P^o^_1/2_)8*s*	^2^[1/2]^o^	1
10 *	25753.37	0.02	3882.987	0.004	2*p*^5^(^2^P^o^_3/2_)4*d*	^2^[7/2]^o^	4	—	2*p*^5^(^2^P^o^_3/2_)6*f*	^2^[7/2]	4
10 *	25753.37	0.02	3882.987	0.004	2*p*^5^(^2^P^o^_3/2_)4*d*	^2^[7/2]^o^	4	—	2*p*^5^(^2^P^o^_3/2_)6*f*	^2^[7/2]	3
10 *	25760.64	0.04	3881.891	0.006	2*p*^5^(^2^P^o^_3/2_)4*d*	^2^[7/2]^o^	3	—	2*p*^5^(^2^P^o^_3/2_)6*f*	^2^[7/2]	3
10 *	25760.64	0.04	3881.891	0.006	2*p*^5^(^2^P^o^_3/2_)4*d*	^2^[7/2]^o^	3	—	2*p*^5^(^2^P^o^_3/2_)6*f*	^2^[7/2]	4
91	25786.665	0.007	3877.9733	0.0010	2*p*^5^(^2^P^o^_3/2_)4*d*	^2^[7/2]^o^	4	—	2*p*^5^(^2^P^o^_3/2_)6*f*	^2^[9/2]	5
85	25794.008	0.003	3876.8694	0.0005	2*p*^5^(^2^P^o^_3/2_)4*d*	^2^[7/2]^o^	3	—	2*p*^5^(^2^P^o^_3/2_)6*f*	^2^[9/2]	4
48 S	25845.550	0.004	3869.1380	0.0005	2*p*^5^(^2^P^o^_3/2_)4*d*	^2^[3/2]^o^	2	—	2*p*^5^(^2^P^o^_3/2_)6*f*	^2^[5/2]	3
130	25861.933	0.003	3866.6870	0.0005	2*p*^5^(^2^P^o^_1/2_)4*s*	^2^[1/2]^o^	1	—	2*p*^5^(^2^P^o^_3/2_)4*p*	^2^[1/2]	0
38	25873.469	0.004	3864.9630	0.0006	2*p*^5^(^2^P^o^_1/2_)4*d*	^2^[5/2]^o^	2	—	2*p*^5^(^2^P^o^_1/2_)6*f*	^2^[7/2]	3
18	25878.656	0.007	3864.1883	0.0010	2*p*^5^(^2^P^o^_3/2_)4*d*	^2^[3/2]^o^	2	—	2*p*^5^(^2^P^o^_3/2_)6*f*	^2^[3/2]	2
56	25879.642	0.003	3864.0411	0.0005	2*p*^5^(^2^P^o^_1/2_)4*d*	^2^[5/2]^o^	3	—	2*p*^5^(^2^P^o^_1/2_)6*f*	^2^[7/2]	4
38	25887.950	0.004	3862.8011	0.0006	2*p*^5^(^2^P^o^_1/2_)4*d*	^2^[3/2]^o^	2	—	2*p*^5^(^2^P^o^_1/2_)6*f*	^2^[5/2]	3
32	25948.961	0.005	3853.7188	0.0007	2*p*^5^(^2^P^o^_3/2_)4*d*	^2^[3/2]^o^	1	—	2*p*^5^(^2^P^o^_3/2_)6*f*	^2^[5/2]	2
21	25960.581	0.007	3851.9939	0.0010	2*p*^5^(^2^P^o^_1/2_)4*d*	^2^[3/2]^o^	1	—	2*p*^5^(^2^P^o^_1/2_)6*f*	^2^[5/2]	2
10 *	25982.412	0.010	3848.7574	0.0014	2*p*^5^(^2^P^o^_3/2_)4*d*	^2^[3/2]^o^	1	—	2*p*^5^(^2^P^o^_3/2_)6*f*	^2^[3/2]	1
10 *	25982.412	0.010	3848.7574	0.0014	2*p*^5^(^2^P^o^_3/2_)4*d*	^2^[3/2]^o^	1	—	2*p*^5^(^2^P^o^_3/2_)6*f*	^2^[3/2]	2
52	26072.823	0.005	3835.4113	0.0008	2*p*^5^(^2^P^o^_3/2_)4*d*	^2^[5/2]^o^	2	—	2*p*^5^(^2^P^o^_3/2_)6*f*	^2^[7/2]	3
68 S	26080.017	0.006	3834.3534	0.0008	2*p*^5^(^2^P^o^_3/2_)4*d*	^2^[5/2]^o^	3	—	2*p*^5^(^2^P^o^_3/2_)6*f*	^2^[7/2]	4
13	26088.520	0.009	3833.1036	0.0013	2*p*^5^(^2^P^o^_3/2_)4*d*	^2^[5/2]^o^	2	—	2*p*^5^(^2^P^o^_3/2_)6*f*	^2^[5/2]	2
17 S	26095.809	0.007	3832.0329	0.0011	2*p*^5^(^2^P^o^_3/2_)4*d*	^2^[5/2]^o^	3	—	2*p*^5^(^2^P^o^_3/2_)6*f*	^2^[5/2]	3
98 *	26131.288	0.003	3826.8301	0.0005	2*p*^5^(^2^P^o^_3/2_)4*f*	^2^[3/2]	2	—	2*p*^5^(^2^P^o^_3/2_)6*g*	^2^[5/2]^o^	3
98 *	26131.288	0.003	3826.8301	0.0005	2*p*^5^(^2^P^o^_3/2_)4*f*	^2^[3/2]	1	—	2*p*^5^(^2^P^o^_3/2_)6*g*	^2^[5/2]^o^	2
15 *	26162.321	0.008	3822.2909	0.0012	2*p*^5^(^2^P^o^_3/2_)4*f*	^2^[9/2]	5	—	2*p*^5^(^2^P^o^_3/2_)6*g*	^2^[9/2]^o^	5
15 *	26162.321	0.008	3822.2909	0.0012	2*p*^5^(^2^P^o^_3/2_)4*f*	^2^[9/2]	4	—	2*p*^5^(^2^P^o^_3/2_)6*g*	^2^[9/2]^o^	4
15 *	26162.321	0.008	3822.2909	0.0012	2*p*^5^(^2^P^o^_3/2_)4*f*	^2^[9/2]	4	—	2*p*^5^(^2^P^o^_3/2_)6*g*	^2^[9/2]^o^	5
230 *	26178.206	0.005	3819.9715	0.0008	2*p*^5^(^2^P^o^_3/2_)4*f*	^2^[9/2]	5	—	2*p*^5^(^2^P^o^_3/2_)6*g*	^2^[11/2]^o^	6
230 *	26178.206	0.005	3819.9715	0.0008	2*p*^5^(^2^P^o^_3/2_)4*f*	^2^[9/2]	4	—	2*p*^5^(^2^P^o^_3/2_)6*g*	^2^[11/2]^o^	5
120 *	26211.856	0.003	3815.0675	0.0004	2*p*^5^(^2^P^o^_1/2_)4*f*	^2^[7/2]	4	—	2*p*^5^(^2^P^o^_1/2_)6*g*	^2^[9/2]^o^	5
120 *	26211.856	0.003	3815.0675	0.0004	2*p*^5^(^2^P^o^_1/2_)4*f*	^2^[7/2]	3	—	2*p*^5^(^2^P^o^_1/2_)6*g*	^2^[9/2]^o^	4
90 *	26213.615	0.003	3814.8115	0.0005	2*p*^5^(^2^P^o^_1/2_)4*f*	^2^[5/2]	2	—	2*p*^5^(^2^P^o^_1/2_)6*g*	^2^[7/2]^o^	3
90 *	26213.615	0.003	3814.8115	0.0005	2*p*^5^(^2^P^o^_1/2_)4*f*	^2^[5/2]	3	—	2*p*^5^(^2^P^o^_1/2_)6*g*	^2^[7/2]^o^	4
110 *	26228.172	0.004	3812.6942	0.0006	2*p*^5^(^2^P^o^_3/2_)4*f*	^2^[5/2]	2	—	2*p*^5^(^2^P^o^_3/2_)6*g*	^2^[7/2]^o^	3
110 *	26228.172	0.004	3812.6942	0.0006	2*p*^5^(^2^P^o^_3/2_)4*f*	^2^[5/2]	3	—	2*p*^5^(^2^P^o^_3/2_)6*g*	^2^[7/2]^o^	4
15 *	26243.848	0.007	3810.4169	0.0010	2*p*^5^(^2^P^o^_3/2_)4*f*	^2^[5/2]	3	—	2*p*^5^(^2^P^o^_3/2_)6*g*	^2^[5/2]^o^	3
15 *	26243.848	0.007	3810.4169	0.0010	2*p*^5^(^2^P^o^_3/2_)4*f*	^2^[5/2]	2	—	2*p*^5^(^2^P^o^_3/2_)6*g*	^2^[5/2]^o^	3
160 *	26276.978	0.003	3805.6126	0.0004	2*p*^5^(^2^P^o^_3/2_)4*f*	^2^[7/2]	4	—	2*p*^5^(^2^P^o^_3/2_)6*g*	^2^[9/2]^o^	5
160 *	26276.978	0.003	3805.6126	0.0004	2*p*^5^(^2^P^o^_3/2_)4*f*	^2^[7/2]	3	—	2*p*^5^(^2^P^o^_3/2_)6*g*	^2^[9/2]^o^	4
25 *	26282.71	0.05	3804.783	0.008	2*p*^5^(^2^P^o^_3/2_)4*f*	^2^[7/2]	3	—	2*p*^5^(^2^P^o^_3/2_)6*g*	^2^[7/2]^o^	3
25 *	26282.71	0.05	3804.783	0.008	2*p*^5^(^2^P^o^_3/2_)4*f*	^2^[7/2]	3	—	2*p*^5^(^2^P^o^_3/2_)6*g*	^2^[7/2]^o^	4
1000	26868.106	0.003	3721.8850	0.0004	2*p*^5^(^2^P^o^_3/2_)4*s*	^2^[3/2]^o^	1	—	2*p*^5^(^2^P^o^_3/2_)4*p*	^2^[1/2]	1
140	27528.250	0.004	3632.6320	0.0006	2*p*^5^(^2^P^o^_1/2_)4*s*	^2^[1/2]^o^	0	—	2*p*^5^(^2^P^o^_3/2_)4*p*	^2^[3/2]	1
930	27580.984	0.003	3625.6865	0.0004	2*p*^5^(^2^P^o^_3/2_)4*p*	^2^[1/2]	0	—	2*p*^5^(^2^P^o^_3/2_)4*d*	^2^[3/2]^o^	1
15	27826.375	0.012	3593.7128	0.0016	2*p*^5^(^2^P^o^_3/2_)4*p*	^2^[3/2]	1	—	2*p*^5^(^2^P^o^_1/2_)5*s*	^2^[1/2]^o^	0
240	27979.570	0.003	3574.0364	0.0004	2*p*^5^(^2^P^o^_3/2_)4*p*	^2^[1/2]	0	—	2*p*^5^(^2^P^o^_3/2_)4*d*	^2^[1/2]^o^	1
570	28393.944	0.003	3521.8778	0.0004	2*p*^5^(^2^P^o^_1/2_)4*p*	^2^[1/2]	0	—	2*p*^5^(^2^P^o^_1/2_)4*d*	^2^[3/2]^o^	1
310	28540.970	0.003	3503.7351	0.0004	2*p*^5^(^2^P^o^_1/2_)4*s*	^2^[1/2]^o^	1	—	2*p*^5^(^2^P^o^_3/2_)4*p*	^2^[3/2]	2
81	28752.113	0.003	3478.0052	0.0004	2*p*^5^(^2^P^o^_1/2_)4*s*	^2^[1/2]^o^	1	—	2*p*^5^(^2^P^o^_3/2_)4*p*	^2^[3/2]	1
13	29295.268	0.006	3413.5206	0.0007	2*p*^5^(^2^P^o^_3/2_)5*p*	^2^[1/2]	1	—	2*p*^5^(^2^P^o^_3/2_)6*d*	^2^[3/2]^o^	2
13	29395.843	0.005	3401.8415	0.0006	2*p*^5^(^2^P^o^_3/2_)5*p*	^2^[1/2]	1	—	2*p*^5^(^2^P^o^_3/2_)6*d*	^2^[1/2]^o^	1
130	29455.855	0.004	3394.9108	0.0004	2*p*^5^(^2^P^o^_3/2_)4*p*	^2^[1/2]	1	—	2*p*^5^(^2^P^o^_3/2_)5*s*	^2^[3/2]^o^	1
14	29495.606	0.003	3390.3355	0.0004	2*p*^5^(^2^P^o^_1/2_)4*p*	^2^[3/2]	1	—	2*p*^5^(^2^P^o^_3/2_)4*d*	^2^[5/2]^o^	2
22	29676.059	0.004	3369.7197	0.0004	2*p*^5^(^2^P^o^_1/2_)4*p*	^2^[3/2]	1	—	2*p*^5^(^2^P^o^_3/2_)4*d*	^2^[3/2]^o^	1
61	29722.119	0.004	3364.4977	0.0004	2*p*^5^(^2^P^o^_1/2_)4*s*	^2^[1/2]^o^	1	—	2*p*^5^(^2^P^o^_3/2_)4*p*	^2^[5/2]	2
6	29812.553	0.004	3354.2917	0.0005	2*p*^5^(^2^P^o^_1/2_)4*p*	^2^[3/2]	1	—	2*p*^5^(^2^P^o^_3/2_)4*d*	^2^[3/2]^o^	2
4	29939.522	0.008	3340.0667	0.0008	2*p*^5^(^2^P^o^_1/2_)4*p*	^2^[3/2]	2	—	2*p*^5^(^2^P^o^_3/2_)4*d*	^2^[5/2]^o^	3
9	29949.038	0.004	3339.0054	0.0004	2*p*^5^(^2^P^o^_1/2_)4*p*	^2^[3/2]	2	—	2*p*^5^(^2^P^o^_3/2_)4*d*	^2^[5/2]^o^	2
1	30127.143	0.014	3319.2659	0.0016	2*p*^5^(^2^P^o^_1/2_)4*p*	^2^[1/2]	1	—	2*p*^5^(^2^P^o^_3/2_)4*d*	^2^[3/2]^o^	1
2	30135.096	0.007	3318.3900	0.0008	2*p*^5^(^2^P^o^_1/2_)4*p*	^2^[3/2]	2	—	2*p*^5^(^2^P^o^_3/2_)4*d*	^2^[3/2]^o^	1
1	30138.002	0.008	3318.0700	0.0009	2*p*^5^(^2^P^o^_1/2_)4*p*	^2^[3/2]	1	—	2*p*^5^(^2^P^o^_3/2_)4*d*	^2^[1/2]^o^	1
10	30173.468	0.017	3314.1699	0.0019	2*p*^5^(^2^P^o^_3/2_)5*p*	^2^[5/2]	3	—	2*p*^5^(^2^P^o^_3/2_)6*d*	^2^[5/2]^o^	3
620	30208.732	0.004	3310.3011	0.0004	2*p*^5^(^2^P^o^_3/2_)4*p*	^2^[1/2]	1	—	2*p*^5^(^2^P^o^_3/2_)5*s*	^2^[3/2]^o^	2
41	30267.823	0.005	3303.8385	0.0005	2*p*^5^(^2^P^o^_1/2_)4*p*	^2^[1/2]	1	—	2*p*^5^(^2^P^o^_3/2_)4*d*	^2^[3/2]^o^	2
17	30275.862	0.004	3302.9613	0.0005	2*p*^5^(^2^P^o^_1/2_)4*p*	^2^[3/2]	2	—	2*p*^5^(^2^P^o^_3/2_)4*d*	^2^[3/2]^o^	2
44	30308.501	0.007	3299.4043	0.0008	2*p*^5^(^2^P^o^_3/2_)5*p*	^2^[5/2	3	—	2*p*^5^(^2^P^o^_3/2_)6*d*	^2^[7/2]^o^	4
20	30371.786	0.008	3292.5295	0.0009	2*p*^5^(^2^P^o^_1/2_)4*p*	^2^[3/2]	2	—	2*p*^5^(^2^P^o^_3/2_)4*d*	^2^[7/2]^o^	3
15	30425.647	0.006	3286.7009	0.0006	2*p*^5^(^2^P^o^_1/2_)5*p*	^2^[3/2]	1	—	2*p*^5^(^2^P^o^_1/2_)6*d*	^2^[5/2]^o^	2
9	30472.757	0.010	3281.6197	0.0011	2*p*^5^(^2^P^o^_3/2_)5*p*	^2^[5/2]	2	—	2*p*^5^(^2^P^o^_3/2_)6*d*	^2^[5/2]^o^	2
12	30475.223	0.006	3281.3542	0.0006	2*p*^5^(^2^P^o^_1/2_)5*p*	^2^[1/2]	1	—	2*p*^5^(^2^P^o^_1/2_)6*d*	^2^[3/2]^o^	2
28	30603.119	0.007	3267.6408	0.0007	2*p*^5^(^2^P^o^_3/2_)5*p*	^2^[5/2]	2	—	2*p*^5^(^2^P^o^_3/2_)6*d*	^2^[7/2]^o^	3
52	30603.346	0.004	3267.6166	0.0004	2*p*^5^(^2^P^o^_1/2_)4*p*	^2^[1/2]	1	—	2*p*^5^(^2^P^o^_3/2_)4*d*	^2^[1/2]^o^	1
21	30639.710	0.005	3263.7385	0.0005	2*p*^5^(^2^P^o^_1/2_)5*p*	^2^[3/2]	2	—	2*p*^5^(^2^P^o^_1/2_)6*d*	^2^[5/2]^o^	3
23	30675.320	0.004	3259.9497	0.0004	2*p*^5^(^2^P^o^_1/2_)4*p*	^2^[1/2]	1	—	2*p*^5^(^2^P^o^_3/2_)4*d*	^2^[1/2]^o^	0
53	30720.023	0.003	3255.2059	0.0004	2*p*^5^(^2^P^o^_3/2_)4*p*	^2^[1/2]	0	—	2*p*^5^(^2^P^o^_1/2_)5*s*	^2^[1/2]^o^	1
16	30928.308	0.008	3233.2839	0.0008	2*p*^5^(^2^P^o^_3/2_)5*p*	^2^[3/2]	1	—	2*p*^5^(^2^P^o^_3/2_)6*d*	^2^[5/2]^o^	2
7	30975.572	0.013	3228.3504	0.0013	2*p*^5^(^2^P^o^_3/2_)5*p*	^2^[3/2]	1	—	2*p*^5^(^2^P^o^_3/2_)6*d*	^2^[3/2]^o^	1
25	31011.644	0.005	3224.5953	0.0005	2*p*^5^(^2^P^o^_3/2_)5*p*	^2^[3/2]	2	—	2*p*^5^(^2^P^o^_3/2_)6*d*	^2^[5/2]^o^	3
11	31110.469	0.008	3214.3521	0.0008	2*p*^5^(^2^P^o^_3/2_)5*p*	^2^[3/2]	2	—	2*p*^5^(^2^P^o^_3/2_)6*d*	^2^[3/2]^o^	2
3	31223.94	0.02	3202.671	0.002	2*p*^5^(^2^P^o^_3/2_)5*p*	^2^[3/2]	2	—	2*p*^5^(^2^P^o^_3/2_)6*d*	^2^[1/2]^o^	1
78	31868.616	0.004	3137.8834	0.0004	2*p*^5^(^2^P^o^_1/2_)4*s*	^2^[1/2]^o^	0	—	2*p*^5^(^2^P^o^_3/2_)4*p*	^2^[1/2]	1
1	32179.408	0.013	3107.5774	0.0012	2*p*^5^(^2^P^o^_3/2_)5*p*	^2^[1/2]	1	—	2*p*^5^(^2^P^o^_3/2_)7*s*	^2^[3/2]^o^	1
7	32433.342	0.005	3083.2469	0.0005	2*p*^5^(^2^P^o^_3/2_)5*p*	^2^[1/2]	1	—	2*p*^5^(^2^P^o^_3/2_)7*s*	^2^[3/2]^o^	2
4	32700.54	0.03	3058.053	0.003	2*p*^5^(^2^P^o^_1/2_)5*p*	^2^[1/2]	0	—	2*p*^5^(^2^P^o^_1/2_)6*d*	^2^[3/2]^o^	1
830	33182.139	0.004	3013.6695	0.0004	2*p*^5^(^2^P^o^_3/2_)4*p*	^2^[5/2]	2	—	2*p*^5^(^2^P^o^_3/2_)5*s*	^2^[3/2]^o^	1
5	33325.251	0.016	3000.7276	0.0014	2*p*^5^(^2^P^o^_3/2_)5*p*	^2^[1/2]	0	—	2*p*^5^(^2^P^o^_3/2_)6*d*	^2^[3/2]^o^	1
230	33341.790	0.004	2999.2391	0.0003	2*p*^5^(^2^P^o^_1/2_)4*p*	^2^[3/2]	1	—	2*p*^5^(^2^P^o^_1/2_)5*s*	^2^[1/2]^o^	1
1700	33361.478	0.004	2997.4691	0.0004	2*p*^5^(^2^P^o^_3/2_)4*p*	^2^[5/2]	3	—	2*p*^5^(^2^P^o^_3/2_)5*s*	^2^[3/2]^o^	2
47	33520.419	0.004	2983.2563	0.0003	2*p*^5^(^2^P^o^_1/2_)4*s*	^2^[1/2]^o^	1	—	2*p*^5^(^2^P^o^_3/2_)4*p*	^2^[1/2]	1
14	33628.670	0.008	2973.6531	0.0007	2*p*^5^(^2^P^o^_3/2_)5*p*	^2^[5/2]	3	—	2*p*^5^(^2^P^o^_3/2_)7*s*	^2^[3/2]^o^	2
2	33686.444	0.015	2968.5532	0.0013	2*p*^5^(^2^P^o^_1/2_)5*p*	^2^[3/2]	1	—	2*p*^5^(^2^P^o^_1/2_)7*s*	^2^[1/2]^o^	1
8	33717.548	0.005	2965.8147	0.0004	2*p*^5^(^2^P^o^_3/2_)5*p*	^2^[5/2]	2	—	2*p*^5^(^2^P^o^_3/2_)7*s*	^2^[3/2]^o^	1
3	33722.372	0.011	2965.3905	0.0009	2*p*^5^(^2^P^o^_1/2_)5*p*	^2^[1/2]	1	—	2*p*^5^(^2^P^o^_1/2_)7*s*	^2^[1/2]^o^	1
3	33813.686	0.016	2957.3824	0.0014	2*p*^5^(^2^P^o^_1/2_)5*p*	^2^[3/2]	1	—	2*p*^5^(^2^P^o^_1/2_)7*s*	^2^[1/2]^o^	0
3	33849.869	0.009	2954.2212	0.0008	2*p*^5^(^2^P^o^_1/2_)5*p*	^2^[1/2]	1	—	2*p*^5^(^2^P^o^_1/2_)7*s*	^2^[1/2]^o^	0
450	33909.054	0.004	2949.0649	0.0004	2*p*^5^(^2^P^o^_1/2_)4*p*	^2^[3/2]	1	—	2*p*^5^(^2^P^o^_1/2_)5*s*	^2^[1/2]^o^	0
440	33912.263	0.004	2948.7858	0.0004	2*p*^5^(^2^P^o^_1/2_)4*p*	^2^[1/2]	1	—	2*p*^5^(^2^P^o^_1/2_)5*s*	^2^[1/2]^o^	1
1200	33922.350	0.004	2947.9090	0.0003	2*p*^5^(^2^P^o^_1/2_)4*p*	^2^[3/2]	2	—	2*p*^5^(^2^P^o^_1/2_)5*s*	^2^[1/2]^o^	1
7	33952.413	0.006	2945.2988	0.0005	2*p*^5^(^2^P^o^_1/2_)5*p*	^2^[3/2]	2	—	2*p*^5^(^2^P^o^_1/2_)7*s*	^2^[1/2]^o^	1
4	33996.462	0.008	2941.4826	0.0007	2*p*^5^(^2^P^o^_3/2_)5*p*	^2^[5/2]	2	—	2*p*^5^(^2^P^o^_3/2_)7*s*	^2^[3/2]^o^	2
360	34140.648	0.004	2929.0598	0.0004	2*p*^5^(^2^P^o^_3/2_)4*p*	^2^[5/2]	2	—	2*p*^5^(^2^P^o^_3/2_)5*s*	^2^[3/2]^o^	2
5	34276.169	0.009	2917.4789	0.0007	2*p*^5^(^2^P^o^_3/2_)5*p*	^2^[3/2]	1	—	2*p*^5^(^2^P^o^_3/2_)7*s*	^2^[3/2]^o^	1
3	34383.050	0.007	2908.4098	0.0006	2*p*^5^(^2^P^o^_3/2_)5*p*	^2^[3/2]	2	—	2*p*^5^(^2^P^o^_3/2_)7*s*	^2^[3/2]^o^	1
590	34480.836	0.004	2900.1617	0.0003	2*p*^5^(^2^P^o^_3/2_)4*p*	^2^[3/2]	1	—	2*p*^5^(^2^P^o^_3/2_)5*s*	^2^[3/2]^o^	1
240	34499.275	0.004	2898.6116	0.0004	2*p*^5^(^2^P^o^_1/2_)4*p*	^2^[1/2]	1	—	2*p*^5^(^2^P^o^_1/2_)5*s*	^2^[1/2]^o^	0
1	34564.44	0.02	2893.1469	0.0018	2*p*^5^(^2^P^o^_3/2_)5*p*	^2^[3/2]	1	—	2*p*^5^(^2^P^o^_3/2_)7*s*	^2^[3/2]^o^	2
6	34673.121	0.007	2884.0784	0.0006	2*p*^5^(^2^P^o^_3/2_)5*p*	^2^[3/2]	2	—	2*p*^5^(^2^P^o^_3/2_)7*s*	^2^[3/2]^o^	2
380	34789.486	0.004	2874.4317	0.0003	2*p*^5^(^2^P^o^_3/2_)4*p*	^2^[3/2]	2	—	2*p*^5^(^2^P^o^_3/2_)5*s*	^2^[3/2]^o^	1
2	35217.80	0.03	2839.473	0.003	2*p*^5^(^2^P^o^_3/2_)5*p*	^2^[1/2]	1	—	2*p*^5^(^2^P^o^_1/2_)5*d*	^2^[3/2]^o^	2
120	35517.017	0.006	2815.5518	0.0004	2*p*^5^(^2^P^o^_3/2_)4*p*	^2^[3/2]	1	—	2*p*^5^(^2^P^o^_3/2_)5*s*	^2^[3/2]^o^	2
790	35844.581	0.004	2789.8220	0.0003	2*p*^5^(^2^P^o^_3/2_)4*p*	^2^[3/2]	2	—	2*p*^5^(^2^P^o^_3/2_)5*s*	^2^[3/2]^o^	2
33	36209.395	0.004	2761.7142	0.0003	2*p*^5^(^2^P^o^_3/2_)3*d*	^2^[1/2]^o^	1	—	2*p*^5^(^2^P^o^_1/2_)4*p*	^2^[1/2]	0
96	36481.630	0.007	2741.1056	0.0005	2*p*^5^(^2^P^o^_1/2_)4*p*	^2^[1/2]	0	—	2*p*^5^(^2^P^o^_3/2_)4*d*	^2^[3/2]^o^	1
3	37176.74	0.02	2689.8538	0.0015	2*p*^5^(^2^P^o^_3/2_)5*p*	^2^[1/2]	0	—	2*p*^5^(^2^P^o^_3/2_)7*s*	^2^[3/2]^o^	1
42	37182.250	0.004	2689.4553	0.0003	2*p*^5^(^2^P^o^_1/2_)4*p*	^2^[1/2]	0	—	2*p*^5^(^2^P^o^_3/2_)4*d*	^2^[1/2]^o^	1
4	37396.549	0.010	2674.0435	0.0007	2*p*^5^(^2^P^o^_3/2_)5*s*	^2^[3/2]^o^	1	—	2*p*^5^(^2^P^o^_1/2_)5*p*	^2^[1/2]	0
130	37746.247	0.004	2649.2700	0.0003	2*p*^5^(^2^P^o^_3/2_)3*d*	^2^[3/2]^o^	1	—	2*p*^5^(^2^P^o^_1/2_)4*p*	^2^[1/2]	0
10	39007.087	0.008	2563.6367	0.0005	2*p*^5^(^2^P^o^_3/2_)4*d*	^2^[1/2]^o^	1	—	2*p*^5^(^2^P^o^_3/2_)5*f*	^2^[5/2]	2
74	39019.950	0.004	2562.7916	0.0005	2*p*^5^(^2^P^o^_3/2_)4*d*	^2^[1/2]^o^	0	—	2*p*^5^(^2^P^o^_3/2_)5*f*	^2^[3/2]	1
170	39136.736	0.005	2555.1441	0.0003	2*p*^5^(^2^P^o^_3/2_)4*d*	^2^[1/2]^o^	1	—	2*p*^5^(^2^P^o^_3/2_)5*f*	^2^[3/2]	2
65	39137.029	0.007	2555.1250	0.0005	2*p*^5^(^2^P^o^_3/2_)4*d*	^2^[1/2]^o^	1	—	2*p*^5^(^2^P^o^_3/2_)5*f*	^2^[3/2]	1
3	39207.468	0.015	2550.5345	0.0010	2*p*^5^(^2^P^o^_3/2_)5*s*	^2^[3/2]^o^	2	—	2*p*^5^(^2^P^o^_1/2_)5*p*	^2^[3/2]	2
66	39324.462	0.005	2542.9464	0.0003	2*p*^5^(^2^P^o^_3/2_)4*d*	^2^[7/2]^o^	4	—	2*p*^5^(^2^P^o^_3/2_)5*f*	^2^[7/2]	4
48	39341.564	0.005	2541.8410	0.0003	2*p*^5^(^2^P^o^_3/2_)4*d*	^2^[7/2]^o^	3	—	2*p*^5^(^2^P^o^_3/2_)5*f*	^2^[7/2]	3
3	39386.615	0.017	2538.9336	0.0011	2*p*^5^(^2^P^o^_3/2_)4*d*	^2^[7/2]^o^	4	—	2*p*^5^(^2^P^o^_3/2_)5*f*	^2^[5/2]	3
4	39403.46	0.05	2537.848	0.003	2*p*^5^(^2^P^o^_3/2_)4*d*	^2^[7/2]^o^	3	—	2*p*^5^(^2^P^o^_3/2_)5*f*	^2^[5/2]	2
640	39457.774	0.004	2534.3548	0.0003	2*p*^5^(^2^P^o^_3/2_)4*d*	^2^[7/2]^o^	4	—	2*p*^5^(^2^P^o^_3/2_)5*f*	^2^[9/2]	5
430	39474.992	0.004	2533.2494	0.0003	2*p*^5^(^2^P^o^_3/2_)4*d*	^2^[7/2]^o^	3	—	2*p*^5^(^2^P^o^_3/2_)5*f*	^2^[9/2]	4
12	39518.767	0.005	2530.4433	0.0003	2*p*^5^(^2^P^o^_3/2_)5*s*	^2^[3/2]^o^	2	—	2*p*^5^(^2^P^o^_1/2_)5*p*	^2^[1/2]	1
260	39566.317	0.004	2527.4023	0.0003	2*p*^5^(^2^P^o^_3/2_)4*d*	^2^[3/2]^o^	2	—	2*p*^5^(^2^P^o^_3/2_)5*f*	^2^[5/2]	3
240	39639.886	0.004	2522.7116	0.0003	2*p*^5^(^2^P^o^_1/2_)4*d*	^2^[5/2]^o^	2	—	2*p*^5^(^2^P^o^_1/2_)5*f*	^2^[7/2]	3
350	39654.346	0.004	2521.7917	0.0003	2*p*^5^(^2^P^o^_1/2_)4*d*	^2^[5/2]^o^	3	—	2*p*^5^(^2^P^o^_1/2_)5*f*	^2^[7/2]	4
280	39669.971	0.004	2520.7984	0.0003	2*p*^5^(^2^P^o^_1/2_)4*d*	^2^[3/2]^o^	2	—	2*p*^5^(^2^P^o^_1/2_)5*f*	^2^[5/2]	3
75	39699.524	0.005	2518.9219	0.0003	2*p*^5^(^2^P^o^_3/2_)4*d*	^2^[3/2]^o^	2	—	2*p*^5^(^2^P^o^_3/2_)5*f*	^2^[3/2]	2
160	39809.128	0.004	2511.9867	0.0003	2*p*^5^(^2^P^o^_3/2_)4*d*	^2^[3/2]^o^	1	—	2*p*^5^(^2^P^o^_3/2_)5*f*	^2^[5/2]	2
180	39817.158	0.004	2511.4801	0.0003	2*p*^5^(^2^P^o^_3/2_)4*p*	^2^[1/2]	0	—	2*p*^5^(^2^P^o^_3/2_)5*s*	^2^[3/2]^o^	1
150	39840.733	0.004	2509.9940	0.0003	2*p*^5^(^2^P^o^_1/2_)4*d*	^2^[3/2]^o^	1	—	2*p*^5^(^2^P^o^_1/2_)5*f*	^2^[5/2]	2
29	39944.171	0.005	2503.4942	0.0003	2*p*^5^(^2^P^o^_3/2_)4*d*	^2^[3/2]^o^	1	—	2*p*^5^(^2^P^o^_3/2_)5*f*	^2^[3/2]	2
20	39944.482	0.005	2503.4747	0.0003	2*p*^5^(^2^P^o^_3/2_)4*d*	^2^[3/2]^o^	1	—	2*p*^5^(^2^P^o^_3/2_)5*f*	^2^[3/2]	1
280	40074.299	0.004	2495.3649	0.0003	2*p*^5^(^2^P^o^_3/2_)4*d*	^2^[5/2]^o^	2	—	2*p*^5^(^2^P^o^_3/2_)5*f*	^2^[7/2]	3
390	40091.275	0.004	2494.3083	0.0003	2*p*^5^(^2^P^o^_3/2_)4*d*	^2^[5/2]^o^	3	—	2*p*^5^(^2^P^o^_3/2_)5*f*	^2^[7/2]	4
65	40138.549	0.005	2491.3706	0.0003	2*p*^5^(^2^P^o^_3/2_)4*d*	^2^[5/2]^o^	2	—	2*p*^5^(^2^P^o^_3/2_)5*f*	^2^[5/2]	2
97	40155.856	0.004	2490.2968	0.0003	2*p*^5^(^2^P^o^_3/2_)4*d*	^2^[5/2]^o^	3	—	2*p*^5^(^2^P^o^_3/2_)5*f*	^2^[5/2]	3
350	40254.424	0.004	2484.1990	0.0003	2*p*^5^(^2^P^o^_3/2_)4*f*	^2^[3/2]	1	—	2*p*^5^(^2^P^o^_3/2_)5*g*	^2^[5/2]^o^	2
540	40254.792	0.004	2484.1763	0.0003	2*p*^5^(^2^P^o^_3/2_)4*f*	^2^[3/2]	2	—	2*p*^5^(^2^P^o^_3/2_)5*g*	^2^[5/2]^o^	3
3	40276.140	0.019	2482.8596	0.0012	2*p*^5^(^2^P^o^_3/2_)4*d*	^2^[5/2]^o^	2	—	2*p*^5^(^2^P^o^_3/2_)5*f*	^2^[3/2]	1
140 *	40291.835	0.004	2481.8924	0.0003	2*p*^5^(^2^P^o^_3/2_)4*f*	^2^[9/2]	4	—	2*p*^5^(^2^P^o^_3/2_)5*g*	^2^[9/2]^o^	4
140 *	40291.835	0.004	2481.8924	0.0003	2*p*^5^(^2^P^o^_3/2_)4*f*	^2^[9/2]	5	—	2*p*^5^(^2^P^o^_3/2_)5*g*	^2^[9/2]^o^	5
6 *	40314.409	0.015	2480.5027	0.0009	2*p*^5^(^2^P^o^_3/2_)4*f*	^2^[9/2]	4	—	2*p*^5^(^2^P^o^_3/2_)5*g*	^2^[7/2]^o^	3
6 *	40314.409	0.015	2480.5027	0.0009	2*p*^5^(^2^P^o^_3/2_)4*f*	^2^[9/2]	5	—	2*p*^5^(^2^P^o^_3/2_)5*g*	^2^[7/2]^o^	4
1900 *	40356.667	0.004	2477.9053	0.0003	2*p*^5^(^2^P^o^_3/2_)4*f*	^2^[9/2]	5	—	2*p*^5^(^2^P^o^_3/2_)5*g*	^2^[11/2]^o^	6
1900 *	40356.667	0.004	2477.9053	0.0003	2*p*^5^(^2^P^o^_3/2_)4*f*	^2^[9/2]	4	—	2*p*^5^(^2^P^o^_3/2_)5*g*	^2^[11/2]^o^	5
1300	40425.964	0.004	2473.6578	0.0003	2*p*^5^(^2^P^o^_1/2_)4*f*	^2^[7/2]	4	—	2*p*^5^(^2^P^o^_1/2_)5*g*	^2^[9/2]^o^	5
660	40429.447	0.007	2473.4447	0.0004	2*p*^5^(^2^P^o^_1/2_)4*f*	^2^[5/2]	2	—	2*p*^5^(^2^P^o^_1/2_)5*g*	^2^[7/2]^o^	3
660 *	40429.447	0.007	2473.4447	0.0004	2*p*^5^(^2^P^o^_1/2_)4*f*	^2^[5/2]	3	—	2*p*^5^(^2^P^o^_1/2_)5*g*	^2^[7/2]^o^	4
300	40429.682	0.008	2473.4303	0.0005	2*p*^5^(^2^P^o^_1/2_)4*f*	^2^[5/2]	3	—	2*p*^5^(^2^P^o^_1/2_)5*g*	^2^[9/2]^o^	4
690	40457.207	0.004	2471.7475	0.0003	2*p*^5^(^2^P^o^_3/2_)4*f*	^2^[5/2]	3	—	2*p*^5^(^2^P^o^_3/2_)5*g*	^2^[7/2]^o^	4
340	40457.474	0.005	2471.7312	0.0003	2*p*^5^(^2^P^o^_3/2_)4*f*	^2^[5/2]	2	—	2*p*^5^(^2^P^o^_3/2_)5*g*	^2^[7/2]^o^	3
85	40522.294	0.007	2467.7774	0.0004	2*p*^5^(^2^P^o^_3/2_)4*f*	^2^[5/2]	3	—	2*p*^5^(^2^P^o^_3/2_)5*g*	^2^[5/2]^o^	3
59 *	40522.553	0.008	2467.7616	0.0005	2*p*^5^(^2^P^o^_3/2_)4*f*	^2^[5/2]	2	—	2*p*^5^(^2^P^o^_3/2_)5*g*	^2^[5/2]^o^	3
59 *	40522.553	0.008	2467.7616	0.0005	2*p*^5^(^2^P^o^_3/2_)4*f*	^2^[5/2]	2	—	2*p*^5^(^2^P^o^_3/2_)5*g*	^2^[5/2]^o^	2
1500 *	40564.426	0.004	2465.2142	0.0003	2*p*^5^(^2^P^o^_3/2_)4*f*	^2^[7/2]	4	—	2*p*^5^(^2^P^o^_3/2_)5*g*	^2^[9/2]^o^	5
1500 *	40564.426	0.004	2465.2142	0.0003	2*p*^5^(^2^P^o^_3/2_)4*f*	^2^[7/2]	3	—	2*p*^5^(^2^P^o^_3/2_)5*g*	^2^[9/2]^o^	4
180 *	40587.308	0.004	2463.8244	0.0003	2*p*^5^(^2^P^o^_3/2_)4*f*	^2^[7/2]	3	—	2*p*^5^(^2^P^o^_3/2_)5*g*	^2^[7/2]^o^	3
180 *	40587.308	0.004	2463.8244	0.0003	2*p*^5^(^2^P^o^_3/2_)4*f*	^2^[7/2]	4	—	2*p*^5^(^2^P^o^_3/2_)5*g*	^2^[7/2]^o^	4
7 *	40652.823	0.012	2459.8538	0.0007	2*p*^5^(^2^P^o^_3/2_)4*f*	^2^[7/2]	3	—	2*p*^5^(^2^P^o^_3/2_)5*g*	^2^[5/2]^o^	2
7 *	40652.823	0.012	2459.8538	0.0007	2*p*^5^(^2^P^o^_3/2_)4*f*	^2^[7/2]	4	—	2*p*^5^(^2^P^o^_3/2_)5*g*	^2^[5/2]^o^	3
10	40818.029	0.008	2449.8978	0.0005	2*p*^5^(^2^P^o^_3/2_)4*f*	^2^[7/2]	4	—	2*p*^5^(^2^P^o^_3/2_)5*d*	^2^[5/2]^o^	3
3	40827.58	0.02	2449.3247	0.0013	2*p*^5^(^2^P^o^_1/2_)4*f*	^2^[5/2]	2	—	2*p*^5^(^2^P^o^_1/2_)5*d*	^2^[3/2]^o^	1
5	40828.183	0.014	2449.2885	0.0008	2*p*^5^(^2^P^o^_3/2_)4*f*	^2^[7/2]	3	—	2*p*^5^(^2^P^o^_3/2_)5*d*	^2^[5/2]^o^	2
2	40851.083	0.012	2447.9155	0.0007	2*p*^5^(^2^P^o^_3/2_)4*f*	^2^[5/2]	2	—	2*p*^5^(^2^P^o^_3/2_)5*d*	^2^[3/2]^o^	1
7	40929.375	0.014	2443.2330	0.0008	2*p*^5^(^2^P^o^_1/2_)4*f*	^2^[7/2]	4	—	2*p*^5^(^2^P^o^_1/2_)5*d*	^2^[5/2]^o^	3
3	40935.11	0.04	2442.891	0.002	2*p*^5^(^2^P^o^_1/2_)4*f*	^2^[7/2]	3	—	2*p*^5^(^2^P^o^_1/2_)5*d*	^2^[5/2]^o^	2
2	40939.10	0.03	2442.6527	0.0016	2*p*^5^(^2^P^o^_1/2_)4*f*	^2^[5/2]	3	—	2*p*^5^(^2^P^o^_1/2_)5*d*	^2^[5/2]^o^	2
2	40941.60	0.04	2442.504	0.002	2*p*^5^(^2^P^o^_1/2_)4*f*	^2^[7/2]	3	—	2*p*^5^(^2^P^o^_1/2_)5*d*	^2^[3/2]^o^	2
3	40945.547	0.019	2442.2680	0.0011	2*p*^5^(^2^P^o^_1/2_)4*f*	^2^[5/2]	3	—	2*p*^5^(^2^P^o^_1/2_)5*d*	^2^[3/2]^o^	2
8	40950.274	0.010	2441.9861	0.0006	2*p*^5^(^2^P^o^_3/2_)4*f*	^2^[9/2]	4	—	2*p*^5^(^2^P^o^_3/2_)5*d*	^2^[7/2]^o^	3
14	40961.383	0.008	2441.3238	0.0005	2*p*^5^(^2^P^o^_3/2_)4*f*	^2^[9/2]	5	—	2*p*^5^(^2^P^o^_3/2_)5*d*	^2^[7/2]^o^	4
8	40992.010	0.015	2439.4998	0.0009	2*p*^5^(^2^P^o^_3/2_)4*f*	^2^[5/2]	3	—	2*p*^5^(^2^P^o^_3/2_)5*d*	^2^[3/2]^o^	2
9	41054.95	0.08	2435.760	0.005	2*p*^5^(^2^P^o^_3/2_)4*f*	^2^[3/2]	2	—	2*p*^5^(^2^P^o^_3/2_)5*d*	^2^[1/2]^o^	1
5	41172.32	0.03	2428.8165	0.0015	2*p*^5^(^2^P^o^_3/2_)5*p*	^2^[1/2]	0	—	2*p*^5^(^2^P^o^_1/2_)5*d*	^2^[3/2]^o^	1
140	42182.976	0.005	2370.6246	0.0003	2*p*^5^(^2^P^o^_1/2_)4*p*	^2^[1/2]	0	—	2*p*^5^(^2^P^o^_1/2_)5*s*	^2^[1/2]^o^	1
5	44335.80	0.02	2255.5134	0.0012	2*p*^5^(^2^P^o^_1/2_)4*p*	^2^[3/2]	1	—	2*p*^5^(^2^P^o^_3/2_)5*s*	^2^[3/2]^o^	1
14	45493.929	0.009	2198.0955	0.0004	2*p*^5^(^2^P^o^_3/2_)3*d*	^2^[1/2]^o^	0	—	2*p*^5^(^2^P^o^_1/2_)4*p*	^2^[1/2]	1
26	45796.921	0.005	2183.5529	0.0003	2*p*^5^(^2^P^o^_3/2_)3*d*	^2^[1/2]^o^	1	—	2*p*^5^(^2^P^o^_1/2_)4*p*	^2^[1/2]	1
27	47159.797	0.006	2120.4502	0.0003	2*p*^5^(^2^P^o^_1/2_)4*p*	^2^[1/2]	1	—	2*p*^5^(^2^P^o^_3/2_)5*s*	^2^[3/2]^o^	2
11	47179.303	0.011	2119.5735	0.0005	2*p*^5^(^2^P^o^_1/2_)4*p*	^2^[3/2]	2	—	2*p*^5^(^2^P^o^_3/2_)5*s*	^2^[3/2]^o^	2
10	47248.165	0.017	2116.4843	0.0008	2*p*^5^(^2^P^o^_3/2_)3*d*	^2^[7/2]^o^	3	—	2*p*^5^(^2^P^o^_1/2_)4*p*	^2^[3/2]	2
9	47588.616	0.014	2101.3429	0.0006	2*p*^5^(^2^P^o^_3/2_)3*d*	^2^[3/2]^o^	2	—	2*p*^5^(^2^P^o^_1/2_)4*p*	^2^[3/2]	2

Letters or symbols in the comment column have the following meanings:

a - asymmetric.

r - self reversed.

* - multiply classified.

S - used as an internal standard for absolute calibration.
